# Simultaneous Determination of 32 Polyphenolic Compounds in Berries via HPLC–MS/MS

**DOI:** 10.3390/molecules30092008

**Published:** 2025-04-30

**Authors:** Yuan Wang, Lijie Xing, Jinlei Zhang, Yongfa Chen, Shiling Lu

**Affiliations:** 1 College of Food Science, Shihezi University, Shihezi 832000, China; duguyuanyuan@126.com; 2 Analysis and Testing Center, Xinjiang Academy of Agriculture and Reclamation Science, Shihezi 832000, China; 15001640887@163.com (L.X.); m202215739303627@163.com (J.Z.); 13070098598@163.com (Y.C.)

**Keywords:** HPLC-MS/MS, berry, polyphenolic compounds, simultaneous determination

## Abstract

An HPLC-MS/MS method for the simultaneous determination of 32 polyphenolic compounds in berries was established. For method validation, the berry samples were extracted with 80% ethanol, purified on an HLB column, and separated on a C18 column via gradient elution with an acetonitrile–water mobile phase system before mass spectrometry detection with electrospray ionization in negative mode and multiple reaction monitoring. The results revealed that the 32 polyphenolic compounds had a good linear relationship in the concentration range of 1–500 μg/L, with R^2^ > 0.99, limits of detection, limits of quantitation, and recoveries of 0.2–0.6 μg/kg, 0.3–1.0 μg/kg, and 82.8–104.8%, respectively, and RSDs < 5.8%. The contents of polyphenolic compounds in the berries were determined, with 23 polyphenolic compounds in sea buckthorn, 18 in mulberry, 17 in black wolfberry, and 12 in red wolfberry. Eight polyphenolic compounds were found in all 4 kinds of berries, including 4-hydroxybenzoic acid, p-coumaric acid, ferulic acid, erucic acid, rutin, hypericin, kaempferol-3-O-rutinoside, and daffinoside. Additionally, six polyphenolic compounds, catechin, syringic acid, isorhamnetin-3-O-galactoside, isorhamnetin-3-O-glucoside, cinnamic acid, and isorhamnetin, were detected only in sea buckthorn.

## 1. Introduction

Berries are a general term for fruits with relatively small sizes and high juice contents, including those of wolfberry, sea buckthorn, and mulberry [1,2,3,4]. Berries are highly appreciated for their sharp color, delicate texture, and unique flavor [5]. Furthermore, these fruits are rich in active ingredients such as polyphenols, flavonoids, and anthocyanins, which can scavenge free radicals in the body, improve immunity, prevent chronic diseases, and exert good health benefits [6,7,8]. Moreover, these fruits have excellent processing properties and have gradually become popular in high-end fruit markets, attracting the attention of consumers and growers alike and becoming the fastest growing emerging fruit tree species.

The extraction and accurate quantification of polyphenolic compounds in berries is an important basis for functional research. The recent research on polyphenols in berries has focused mainly on their extraction with solvents including water [9], methanol [10], ethanol [11], or mixtures of water and methanol [12] or ethanol [13]. The extraction of polyphenolic compounds from plants by an ethanol–water solution is widely used. Qian et al. [14] compared the extraction efficiency of flavonoids in jujube berries using 100% water, 50% ethanol–water, and 90% ethanol–water as extraction solvents and reported that the yield was highest with 90% ethanol. Stephen Inbaraj et al. [15] used 50% ethanol as the extraction solvent and analyzed 15 phenolic acids and flavones in wolfberry via HPLC-MS/MS.

The current methods for determining phenolic compounds include mainly high-performance liquid chromatography (HPLC) [16,17,18], gas chromatography–mass spectrometry (GC-MS) [19,20], and liquid chromatography–mass spectrometry (LC-MS) [21,22,23]. HPLC is often used for the analysis of phenolic compounds but is limited by interference from complex matrices when analyzing multiple compounds. MS can provide precise structural information, and if the compounds have different molecular weights, liquid chromatography coelution is not a serious problem. GC-MS can provide good resolution, but the necessary derivatization step is complicated and time-consuming. Liquid chromatography–time of flight mass spectrometry (LC-TOF/MS) is often used for the screening and identification of active components in plants [24,25], but there are few studies on the quantitative analysis of compounds, especially in multi-group determination [26]. Among the above-mentioned methods, the HPLC method has poor qualitative performance and a long analysis time. The GC-MS method requires derivatization and the steps are cumbersome. The LC-TOF/MS method is mostly used for the qualitative analysis of compounds. High-performance liquid chromatography–tandem triple quadrupole mass spectrometry (HPLC–MS/MS) has strong resistance to interference and high sensitivity, allowing for accurate qualitative and quantitative analyses. Through the optimization of conditions such as the analytical chromatographic column, mobile phase, extraction solvent, and solid-phase extraction column, the problems of poor qualitative analysis and low sensitivity in the process of polyphenol analysis were solved.

Black wolfberry, red wolfberry, mulberry and sea buckthorn, as representative products of berries, have high economic value and are often used in the development of functional products. These berries have been identified as containing a variety of polyphenolic compounds, including more than 30 kinds such as rutin, caffeic acid, and ferulic acid, which play an important role in human health. In this study, we aimed to establish an HPLC–MS/MS for the determination of 32 phenolic compounds in berries with the goal of providing a technique for the functional study of phenolic compounds in berries.

## 2. Results

### 2.1. Optimization of the MS Parameters

The standard solution of each individual polyphenol was diluted to 1 μg/mL, the MS/MS conditions were optimized by direct injection of the working solution, and the fragment parameters were adjusted to maximize the signal response of the precursor ion. Furthermore, adjustment of the collision energy (CE) allowed optimization of the response values of the product ions, and the product ion with the highest response value was selected as the quantitative ion, while the ion with the second highest response value was selected as the qualitative product ion (Table 1 and Figure S1).

### 2.2. Effects of Different Chromatographic Columns on Polyphenol Separation

**Figure 1:**
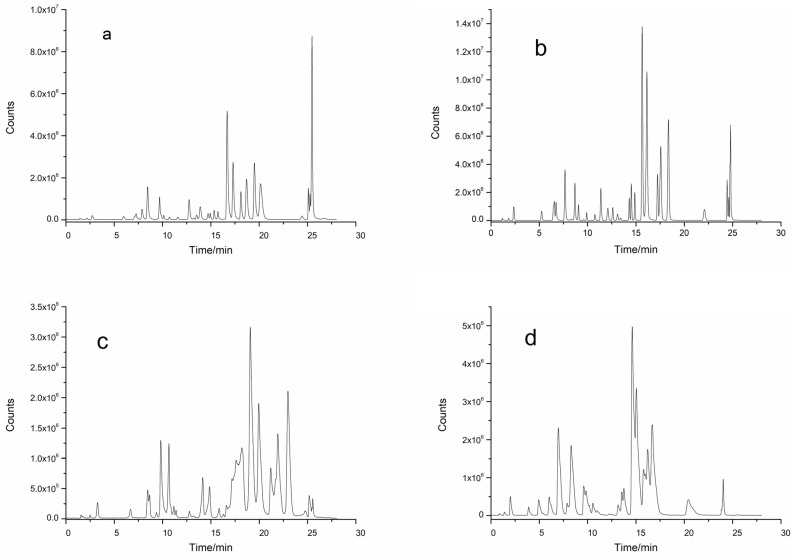
TIC of different chromatographic columns on the separation of phenolic compounds by HPLC-MS/MS. (**a**) ZORBAX Extend-C18 column; (**b**) Infinity Lab Poroshell 120 EC-C18 column; (**c**) ZORBAX SB-C18 column; (**d**) ZORBAX SB-Aq C18 column.

The abilities of four analytical chromatographic columns, a ZORBAX Extend-C18 column (3.0 × 100 mm, 1.8 μm), an InfinityLab Poroshell 120 EC-C18 column (3.0 × 100 mm, 2.7 μm), a ZORBAX SB-C18 column (3.0 × 100 mm, 1.8 μm), and a ZORBAX SB-Aq C18 column (3.0 × 100 mm, 3.5 μm), to separate 32 phenolic compounds were investigated by HPLC-MS/MS. The results revealed that the total ion chromatography (TIC) of ZORBAX Extend-C18 (Figure 1a), ZORBAX SB-C18 (Figure 1c), and ZORBAX SB-Aq (Figure 1d) columns had poor separation efficiency and low component response values and exhibited peak trailing when analyzing the 32 polyphenolic compounds. However, the InfinityLab Poroshell 120 EC-C18 (Figure 1b) column effectively separated 32 polyphenolic compounds with high component response values.

### 2.3. Effects of Different Mobile Phases on Polyphenol Separation

**Figure 2:**
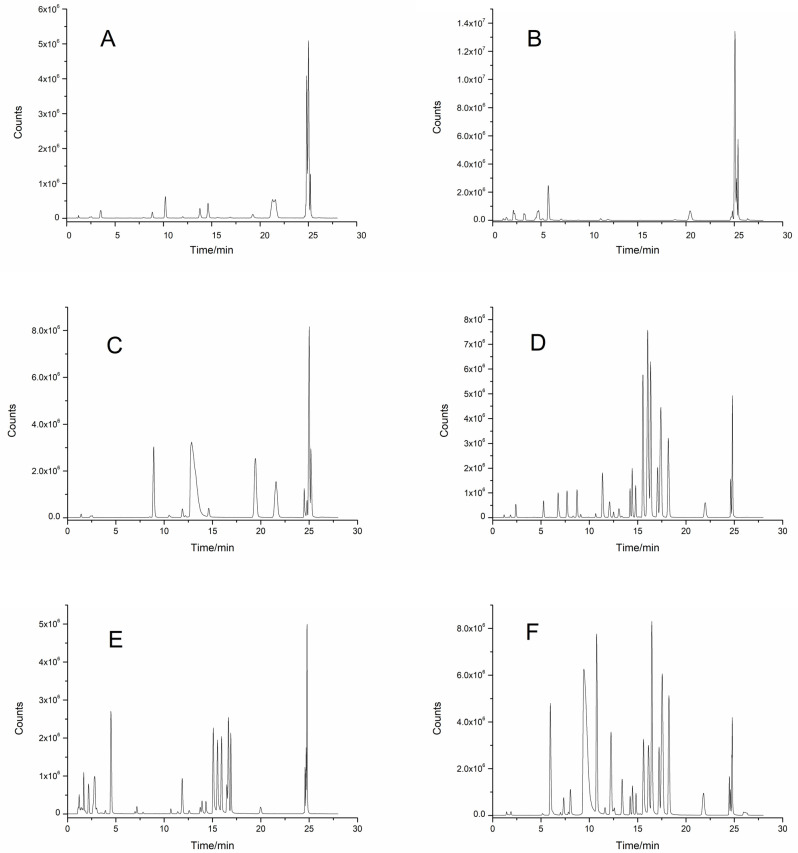
TIC of different mobile phases on the separation of phenolic compounds in InfinityLab Poroshell 120 EC-C18 column by HPLC-MS/MS. (**A**) Methanol–water; (**B**) methanol-0.1% formic acid water; (**C**) methanol-5 mmol/L ammonium acetate solution; (**D**) acetonitrile–water; (**E**) acetonitrile-0.1% formic acid water; (**F**) acetonitrile-5 mmol/L ammonium acetate solution.

The separation effects of six different mobile phase systems, methanol–water, methanol-0.1% aqueous formic acid, methanol-5 mmol/L ammonium acetate, acetonitrile–water, acetonitrile-0.1% aqueous formic acid, and acetonitrile-5 mmol/L ammonium acetate, were compared by HPLC-MS/MS. The results showed that when methanol–water (Figure 2A), methanol-0.1% aqueous formic acid (Figure 2B), and methanol-5 mmol/L ammonium acetate (Figure 2C) were used as mobile phases the separation effects and responses of 32 polyphenols were poor. When acetonitrile–water (Figure 2D), acetonitrile–0.1% aqueous formic acid (Figure 2E), and acetonitrile-5 mmol/L ammonium acetate (Figure 2F) were used as the mobile phases the response values of the polyphenolic compounds were greater, and the peak shapes were more symmetrical. In addition, the addition of formic acid and ammonium acetate to the mobile phase of the acetonitrile system did not improve the response or peak pattern of the polyphenolic compounds. When acetonitrile–water was used as the mobile phase (Figure 2D), the degree of separation and the response values of 32 polyphenolic compounds were the highest, so acetonitrile–water was selected as the mobile phase to analyze 32 polyphenolic compounds in berries.

### 2.4. Effects of Different Solvents on Extraction Efficiency

**Figure 3:**
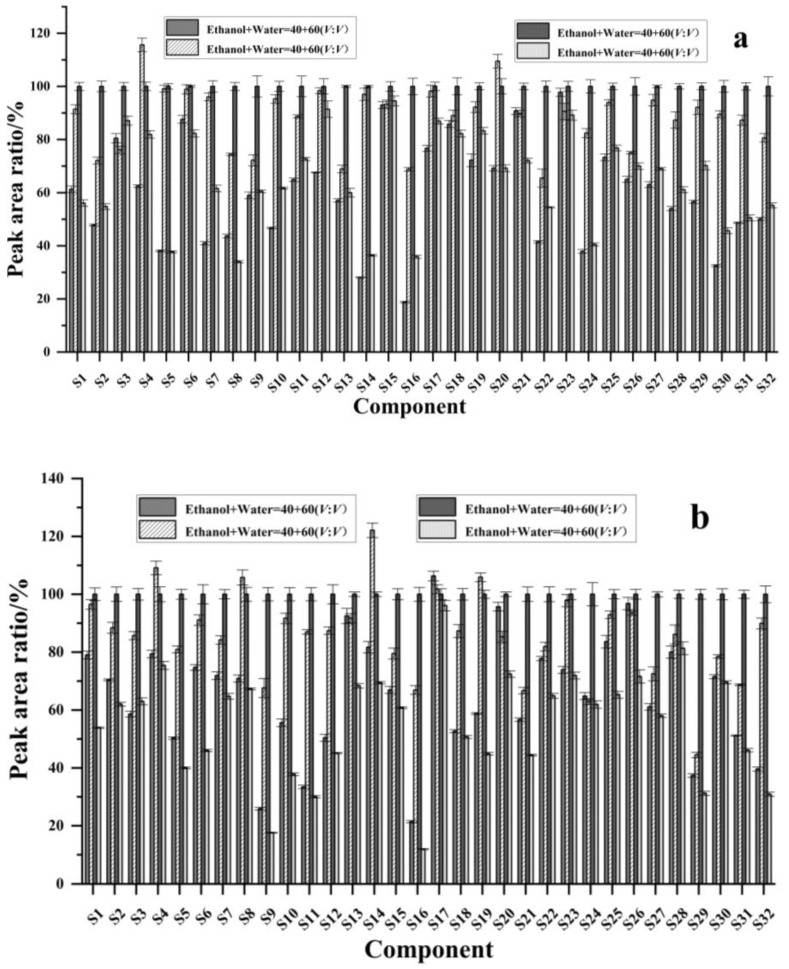

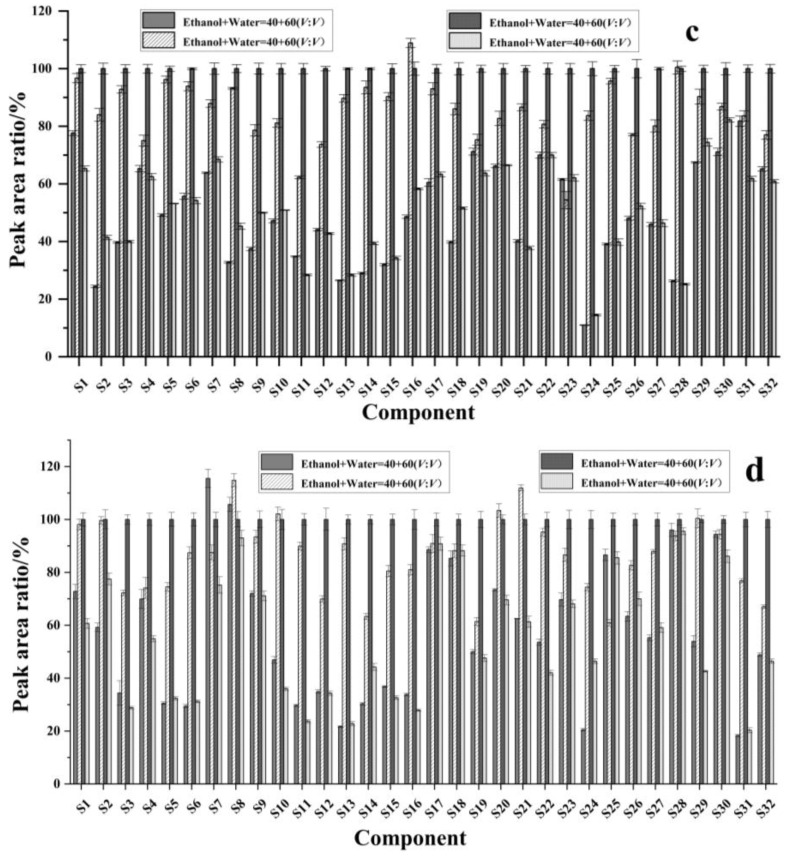
Effects of different extraction solvents on the extraction efficiency of phenolic compounds (**a**) black wolfberry; (**b**) red wolfberry; (**c**) mulberry; (**d**) sea buckthorn. S1: arbutin, S2: gallic acid, S3: 4-hydroxybenzoic acid, S4: epigallocatechin, S5: neochlorogenic acid, S6: D-quinic acid, S7: catechin, S8: caffeic acid, S9: syringic acid, S10: epicatechin, S11: p-coumaric acid, S12: vanilloid acid, S13: 7-hydroxycoumaric acid, S14: chlorogenic acid, S15: ferulic acid, S16: sinapic acid, S17: rutin, S18: isoferulic acid, S19: hyperoside, S20: isoquercitrin, S21: quercetin 3-O-β-D-xylopyranoside, S22: guaijaverin, S23: salicylic acid, S24: kaempferol 3-rutinoside, S25: narcissin, S26: astragalin, S27: isorhamnetin 3-O-galactoside, S28: isorhamnetin 3-O-glucoside, S29: phloridin, S30: cinnamic acid, S31: naringin, and S32: isorhamnetin.

Taking the response peak area of each component upon extraction with 80% ethanol as references, the response peak area of each component and its ratio upon extraction with other solvents were plotted as the vertical coordinate, with the component name on the horizontal coordinate. As shown in Figure 3, when anhydrous ethanol was used as the solvent, the response peak area ratios of black wolfberry ranged from 34.0% to 94.5%, among which 19 components had ratios less than 70%; the response peak area ratios of red wolfberry ranged from 17.6% to 96.1%, and 26 components had ratios less than 70%; the response peak area ratios of mulberry ranged from 14.5% to 82.2%, and 30 components had ratios less than 70%; and the response peak area ratios of sea buckthorn ranged from 20.3% to 95.5%, and 23 components had ratios less than 70%. When 40% ethanol was used as the solvent, the response peak area ratios of *L. barbarum* ranged from 18.8% to 97.8%, and 23 components had ratios less than 70%; the response peak area ratios of *L. barbarum* ranged from 21.4% to 106.3%, and 16 components had ratios less than 70%; the response peak area ratios of mulberry were between 11.0% and 81.8%, and 28 components had ratios less than 70%; and the response peak area ratios of sea buckthorn ranged from 21.7% to 115.5%, of which 22 components had a ratio less than 70%. When 60% ethanol was used as the solvent, the response peak area ratios of black wolfberry ranged from 65.5% to 115.6%, and there were two components with ratios less than 70%; the response peak area ratios of red wolfberry ranged from 44.5% to 122.1%, and five components had ratios less than 70%; the response peak area ratios of mulberry ranged from 54.4% to 108.9%, and two components had ratios less than 70%; and the response peak area ratios of sea buckthorn ranged from 60.9% to 111.9%, and five components had ratios less than 70%. In summary, the extraction efficiencies of the 40% ethanol solution and pure ethanol solution were low, whereas the extraction efficiencies of 60% ethanol and 80% ethanol were high, with the greatest extraction efficiency observed with 80% ethanol. These results may be because the polyphenolic compounds in the berries more easily dissolve in solutions with a high proportion of ethanol.

### 2.5. Effects of Different Solid-Phase Extraction Columns on Purification

**Figure 4:**
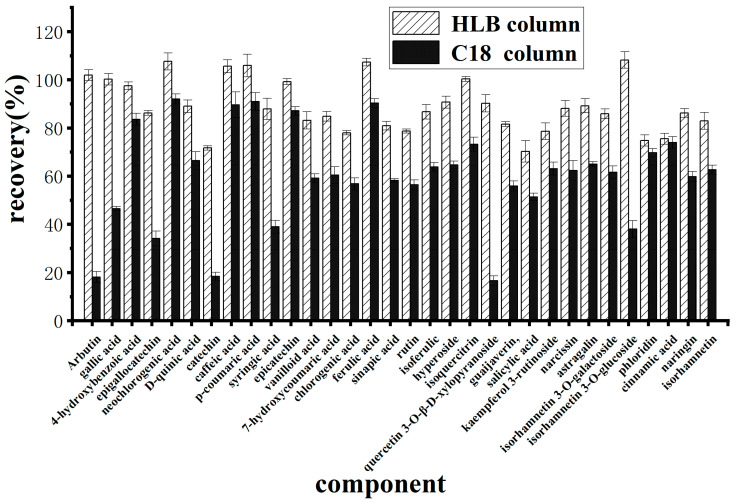
Effects of different solid phase extraction columns on purification.

Owing to the complexity and high sugar content of the berry matrix, the use of a solid-phase extraction column to remove matrix interference aids in the accurate quantification of polyphenolic compounds in a sample. To ensure good adsorption of polyphenolic compounds to the HLB and C18 solid-phase extraction columns, an acidic aqueous solution was used to dissolve the polyphenolic compounds because they contain phenolic hydroxyl groups. The results (Figure 4) revealed that when a C18 column was used for purification, the recoveries of arbutin, gallic acid, epigallocatechin, catechin, eugenic acid, isoquercetin-3-O-β-xyloside, and isorhamnose-3-O-glucoside were all less than 50%. However, when the HLB column was used, the recoveries of the 32 polyphenolic compounds ranged from 70.3% to 108.2%.

### 2.6. Method Validation

#### 2.6.1. Specificity, Linear Range, Limit of Detection (LOD), and Limit of Quantification (LOQ)

Owing to the presence of isomers among polyphenolic components, accurate qualitative and quantitative data can be obtained only after optimizing the chromatographic conditions to ensure effective separation. A series of standard mixed solutions containing 32 polyphenolic compounds were prepared as described in Section 4.3 and analyzed under the optimized instrumental conditions. As shown in Table 2, each of the 32 polyphenolic compounds showed a good linear relationship within the corresponding linear range, with correlation coefficients R^2^ > 0.99. The LOD was 0.2–1.0 μg/kg, and the LOQ was 0.6–3.0 μg/kg.

#### 2.6.2. Accuracy and Precision

To evaluate the accuracy and precision of the method developed to determine phenolic compounds in samples, each sample was analyzed six consecutive times under the optimized instrumental and pretreatment conditions: these results are shown in Table 3. The recoveries of the 32 polyphenolic compounds were in the range of 82.8–104.8%, with relative standard deviations (RSDs) < 5.8%, indicating that the established method had good adaptability and could be used for the rapid qualitative and quantitative determination of polyphenolic compounds in berry samples.

### 2.7. Quantitative Analysis of Polyphenolic Compounds in 4 Kinds of Berries Found in Xinjiang

To verify the effectiveness and applicability of the developed method, the polyphenolic compounds in samples of black wolfberry (Figure S2A), red wolfberry (Figure S2D), mulberry (Figure S2C), and sea buckthorn (Figure S2B) were analyzed under the optimized experimental conditions (Table 4). The results revealed the presence of 23 polyphenols in sea buckthorn, with a total polyphenolic content of 634.56 mg/kg. Additionally, 18 polyphenols were detected in mulberry with a total polyphenolic content of 515.55 mg/kg; 17 polyphenols were detected in *L. barbarum* with a total polyphenolic content of 324.16 mg/kg; and 12 polyphenols were detected in red wolfberry with a total polyphenolic content of 299.64 mg/kg. Eight polyphenolic compounds were detected in all four kinds of berries, including 4-hydroxybenzoic acid, p-coumaric acid, ferulic acid, erucic acid, rutin, hypericin, kaempferol-3-O-rutinoside, and daffinoside. Six polyphenolic compounds, catechin, syringic acid, isorhamnetin-3-O-galactoside, isorhamnetin-3-O-glucoside, cinnamic acid, and isorhamnetin, were detected only in sea buckthorn.

## 3. Discussion

The type of analytical column plays a crucial role when separating many compounds. Owing to the structural characteristics of polyphenolic compounds, most of the 32 polyphenolic compounds in this study contain hydroxyl and carboxyl groups and some are isomers of each other, so their separation directly affects the subsequent qualitative and quantitative analyses. C18 columns are preferred for the separation of polyphenols because of their wide polarity coverage, excellent selectivity, gradient compatibility, and stability [24]. The Poroshell 120 EC-C18 column uses surface porous particles (core–shell structure) in which the thickness of the porous layer is only one-third that of fully porous fillers, shortening the mass transfer path and significantly improving column efficiency, which is particularly suitable for separating polyphenolic isomers with similar structures [25]. Since these 32 polyphenols are mainly of strong and medium polarity, a high proportion of a polar solvent, such as 50% to 80% methanol [16] or ethanol aqueous solutions [26], was selected for extraction. Ethanol is often chosen because of its lower toxicity and good polyphenol solubility. Atanacković Krstonošić et al. [27] used a Poroshell 120 EC-C18 column to separate 16 phenolic compounds from wine samples, and the separation effect was good, and the total running time was 30 min. The C18 bonding phase of this column is end-capped, which effectively shields the residual silicone hydroxyl group on the surface of the silica gel, reduces the peak tailing phenomenon of the polyphenols, and improves the peak shape, making this column the best choice for polyphenol analysis. In contrast, the high pH tolerance of ZORBAX Extend-C18, the strong polarity compatibility of SB-Aq, and the low pH stability of SB-C18 have advantages in specific scenarios, but the overall polyphenol separation performance of these columns was not as good as that of the InfinityLab Poroshell 120EC-C18 column.

When analyzing polyphenolic compounds by HPLC-MS/MS, the mobile phase directly affects the separation efficiency, peak shape, and detection sensitivity. Mobile phases usually include a methanol system (methanol–water/formic acid/ammonium acetate) [28] and an acetonitrile system (acetonitrile–water/formic acid/ammonium acetate) [29,30]. As shown in Figure 2, poor separation was achieved with the methanol system, along with severe overlap of the polyphenol peaks, low response values, and asymmetric peak shapes. With the acetonitrile system, the degree of separation was significantly better, the response values of 32 polyphenolic compounds increased, and the peak symmetry improved. The elution strength of acetonitrile was greater than that of methanol, and the viscosity of the acetonitrile–water system was significantly lower than that of the methanol–water system. Using acetonitrile in the mobile phase leads to the faster elution of hydrophobic compounds, shortening the difference in retention time between the polar polyphenols and hydrophobic polyphenols and avoiding coeluting peaks. Moreover, the response values and peak shapes of the polyphenols did not significantly improve after the addition of formic acid or ammonium acetate to the acetonitrile system. Because the polyphenolic compounds were analyzed in negative ion mode, the addition of formic acid inhibited their ionization efficiency [31].

The extraction efficiency of polyphenols from berries depends on the extraction solvent. Owing to the chemical properties of polyphenolic compounds, extraction solvents commonly used include methanol, ethanol, water, etc. Mustafa [26] used ethanol–water mixture (70:30, *v*/*v*) to extract 36 phenolic compounds (7 anthocyanins, 9 flavonols, 4 flavan-3-ols, 2 dihydrochalcones, 2 flavanones and 12 phenolic acids) from blueberries and strawberries, and the extraction efficiency was high. The results of this study showed that ethanol could be used to extract polyphenolic compounds from berries, and the proportion of ethanol directly affected the extraction efficiency. The extraction efficiency of polyphenolic compounds in berries using different ethanol–water mixtures as the extraction solvent was compared. Ethanol, 40% ethanol–water solution, 60% ethanol–water solution, and 80% ethanol–water solution were added to the berry samples for extraction. Then, the extract was concentrated, and the sample solution was purified by an HLB column and analyzed by HPLC-MS/MS. The results show that the extraction efficiency of 80% ethanol–water solution as the extraction solvent is generally the best, which is higher than that of 60% ethanol–water solution, 40% ethanol–water solution, and ethanol.

Owing to the complexity and high sugar content of the berry matrix, the use of a solid-phase extraction column to remove matrix interference aids in the accurate quantification of polyphenolic compounds in a sample. Most polyphenolic compounds contain phenolic hydroxyl groups. To ensure good adsorption of polyphenolic compounds to HLB and C18 solid-phase extraction columns, an acidic aqueous solution was used to dissolve the polyphenolic compounds. The results revealed that the retention of 32 polyphenols on the HLB column was greater than that on the C18 column. This occurred because these 32 phenolic compounds are mainly of strong and intermediate polarity. The HLB column consists of a hydrophilic lipophilic equilibrium copolymer (n-vinylpyrrolidone and divinylbenzene) that can retain both polar and nonpolar compounds. The C18 column relies mainly on hydrophobic interactions and is suitable for use with nonpolar or weakly polar compounds but has poor retention of strongly polar compounds. Stephen Inbaraj et al. [15] used a Phenomenex Strata-X cartridge solid-phase extraction column (similar to HLB packing) to determine polyphenolic compounds in wolfberry by HPLC-DAD-ESI-MS, and results were satisfactory. Therefore, an HLB column is more suitable for the purification of polyphenols.

Compared with the literature methods for the determination of polyphenolic compounds (Table 5), the results showed the good separation effect of HPLC when a few polyphenols were analyzed. However, in the presence of many components, some of the components do not separate from the baseline and multiwavelength determination is needed, which is not conducive to quantification and long run times. Moreover, the sensitivity of the determination of polyphenolic compounds by the liquid-phase method is low, and there is a possibility of false positive results when considering only the retention time. GC-MS usually requires derivatization of compounds to determine, and the steps are complicated. LC-TOF is often used for the screening and identification of active components in plants, but there are few studies on the quantitative analysis of compounds. Thus, liquid chromatography–tandem high-resolution mass spectrometry is generally used for compound identification. When analyzing polyphenols in plants, liquid chromatography–tandem triple quadrupole mass spectrometry has the advantages of rapid quantitative analysis, high sensitivity, and good accuracy; therefore, this technique is often used for the analysis of multicomponent mixtures. Meanwhile, this method has good separation and high sensitivity, which can provide ideas for the development of anthocyanin determination methods in berries. Due to the chemical properties and structural characteristics of anthocyanins, especially the color of anthocyanins, when analyzing polyphenolic compounds the extraction and purification of anthocyanins, as an important class of phenolic compounds in black wolfberries and mulberries, are different from those of flavonoids and phenolic acids. Therefore, a separate LC-MS/MS determination method was developed for anthocyanins in berries. Compared with the reported polyphenolic compounds determination methods, this method significantly increases the number of analyzed polyphenolic compounds and improves the response of polyphenol compounds through the optimization of the mobile phase, chromatographic column, extraction solvent, and solid-phase extraction column. In addition, the InfinityLab Poroshell 120 EC-C18 column was adopted to overcome the previous difficulty in separating isomers.

4-hydroxybenzoic acid, p-coumaric acid, ferulic acid, erucic acid, rutin, hypericin, kaempferol-3-O-rutinoside, and isorhamnetin often exist in berries, and most of them have antioxidant, anti-inflammatory, and neuroprotective effects. In this study, the sum of the single polyphenol content determined by HPLC-MS/MS is significantly different from the total phenol content determined by spectrophotometry, mainly because spectrophotometry is non-specific and easily interfered with by other substances. The content is usually higher than that determined by chromatography or mass spectrometry. Zhao et al. [46] used UPLC–MS/MS to detect nine polyphenolic compounds in Gansu black wolfberry, in which the contents of rutin and isoquercetin were relatively high. Zhang et al. [47] used UPLC-Q-Orbitrap MS to detect 18 polyphenolic compounds in Qinghai wolfberry, in which the contents of chlorogenic acid and rutin were relatively high. Gundogdu et al. [43] used HPLC to detect nine polyphenolic compounds in Turkey mulberry, and the contents of rutin and gallic acid were relatively high. Guo et al. [31] quantitatively analyzed 16 polyphenolic compounds in sea buckthorn by HPLC and found that the content of isorhamnetin-3-rutinoside was the highest. The types and contents of polyphenolic compounds in berries reported are inconsistent with the results determined by this method, which is due to the unique climate of Xinjiang. Ju et al. [48] used UPLC-IM-QTOF-MS to screen and quantify polyphenolic compounds in Xinjiang wolfberry. The results showed that 11 polyphenolic compounds were detected in Xinjiang wolfberry, and the highest content of rutin was 125.38 µg/g, which was consistent with the results of this study.

## 4. Materials and Methods

### 4.1. Reagents and Materials

Acetonitrile and methanol were LC-MS grade reagents purchased from Fischer Corporation (Fisher, Dreieich, Germany). Ethyl alcohol, ammonium acetate, and formic acid were HPLC grade reagents purchased from CNW Corporation (CNW, Remscheid, Germany). Ultrapure water (MilliporeSigma, Billerica, MA, USA) was used in all experiments. The C18 and HLB SPE columns (60 mg/3 mL) were purchased from Waters Corporation (Waters, Milford, MA, USA). Black wolfberry, red wolfberry, mulberry, and sea buckthorn were purchased from a local market in Xinjiang.

### 4.2. Instruments and Equipment

A 1200 + 6460 high-performance liquid chromatograph–tandem triple quadrupole mass spectrometer (Agilent, Santa Clara, CA, USA) was used in this study, along with an MS3-basic vortex mixer (IKA, Staufen, Germany), a 3-30K centrifuge (Sigma, Osterode, Germany), a 112 Nitrogen Evaporator (Organomation, Jersey, NJ, USA), ME2002E and ME203E electronic balances (Mettler Toledo, Zurich, Switzerland), an SBL-30DT ultrasonic constant temperature cleaner (Ningbo Scientz Biotechnology, Ningbo, China), and an IQ7000 Milli-Q water system (MilliporeSigma, Billerica, MA, USA).

### 4.3. Preparation of Standard Solutions

The arbutin, gallic acid, 4-hydroxybenzoic acid, epigallocatechin, neochlorogenic acid, D-quinic acid, catechin, caffeic acid, syringic acid, epicatechin, p-coumaric acid, vanilloid acid, 7-hydroxycoumaric acid, chlorogenic acid, ferulic acid, sinapic acid, rutin, isoferulic acid, hyperoside, isoquercitrin, quercetin 3-O-β-D-xylopyranoside, guaijaverin, salicylic acid, kaempferol 3-rutinoside, narcissin, astragalin, isorhamnetin 3-O-galactoside, isorhamnetin 3-O-glucoside, phloridin, cinnamic acid, naringin, and isorhamnetin standards were obtained from Sigma (Sigma-Aldrich, Steinheim, Germany) with purities greater than 95%. Each individual polyphenol standard was dissolved in anhydrous ethanol to generate 1.00 mg/mL solutions, which were stored at −20 °C. A 10.0 µg/mL mixed standard solution was prepared by diluting the 32 individual standard solutions with methanol and stored at −4 °C.

### 4.4. HPLC-MS/MS Conditions

#### 4.4.1. Chromatographic Conditions

An InfinityLab Poroshell 120 EC-C18 column (3.0 × 150 mm, 2.7 μm) was used for the separation of the 32 polyphenolic compounds, with a column temperature of 30 °C and injection volume of 5 µL. Chromatographic separation was carried out via gradient elution with mobile phases A (acetonitrile) and B (water) at a flow rate of 0.40 mL/min as follows: 0–5 min, 5–10% A; 5–8 min, 10–15% A; 8–11 min, 15% A; 11–12 min, 15–18% A; 12–22 min, 18%; 22–23 min, 18–95% A; and 23–28 min, 95% A. The total chromatographic run time was 28 min.

#### 4.4.2. Mass Spectrometry Conditions

Electrospray ionization was performed in negative ion mode (ESI-) with the following parameters: multiple reaction monitoring (MRM) mode; capillary voltage, 3.5 kV; dry gas flow rate, 10 L/min; dry temperature, 325 °C; sheath temperature, 350 °C; sheath gas flow rate, 12 L/min; and atomizer pressure, 45 psi.

### 4.5. Sample Pretreatment

Each berry sample (2.00 ± 0.05 g) was weighed into a 50 mL plastic centrifuge tube, and 15 mL of ethanol solution (ethanol:water 80:20, *v*/*v*) was added, after which the mixture was mixed for 30 s, extracted in an ultrasonic apparatus for 10 min, and centrifuged at 8000 r/min for 5 min before the supernatant was collected. The extraction procedure was repeated, and the supernatants were combined. The solution was then concentrated to 1 mL, 5.0 mL of 0.01 mol/L HCl solution was added at 40 °C, and the solution was mixed well.

The HLB solid-phase extraction column was first washed with 5 mL of methanol and then with 5 mL of 0.01 mol/L aqueous HCl, after which the pretreated solution was added. The column was then washed with 5 mL of water before sample elution with 5 mL of ammoniated methanol. The sample solvent was removed with a stream of nitrogen in a water bath at 40 °C, and the residue was dissolved in 1.0 mL of methanol–water (1:1, *v*/*v*), filtered through a 0.22 µm organic syringe filter, and analyzed via HPLC–MS/MS. If the concentration of phenolic compounds in the sample exceeded the linear range, the solution was further diluted with methanol–water (1:1, *v*/*v*) to obtain peaks in the linear range.

### 4.6. Method Validation

The specificity, linear range, LOD, LOQ, accuracy, and precision of the established method were evaluated. The intermediate solution was diluted stepwise to generate a series of mixed standard solutions with concentrations of 1000.0 ng/mL, 500.0 ng/mL, 200.0 ng/mL, 100.0 ng/mL, 50.0 ng/mL, 20.0 ng/mL, 10.0 ng/mL, 5.0 ng/mL, and 2.0 ng/mL. After analysis under the optimized experimental conditions, the data were plotted with the concentration as the horizontal coordinate and the response as the vertical coordinate, and the linear relationship was evaluated via linear regression. The LODs and LOQs were calculated for each compound as signal-to-noise ratios of 3 and 10, respectively. Considering that the berry sample itself contains polyphenolic compounds, the mixed standard solution (500 µg/kg) was added to the sample, the sample was analyzed 6 times, and the recovery rate and RSD of each component were calculated to evaluate the accuracy and precision of the method.

### 4.7. Data Processing

All the experimental results are presented as the means ± standard deviations of three parallel measurements. All the images were created with Origin 2021 software.

## 5. Conclusions

An HPLC–MS/MS method was developed for the rapid quantitative analysis of 32 polyphenolic compounds in berry samples. The berry samples were extracted with 80% ethanol and purified with an HLB solid-phase extraction column. The samples were then separated on an InfinityLab Poroshell 120 EC-C18 column. Acetonitrile–water was used as the mobile phase with gradient elution. The 32 polyphenols had a good linear relationship in their corresponding linear range, with correlation coefficients R^2^ > 0.99. The LODs ranged from 0.2 to 1.0 μg/kg, the LOQs ranged from 0.6 to 3.0 μg/kg, the recoveries ranged from 82.8 to 104.8%, and the RSDs were <5.8%. The polyphenol contents of the characteristic Xinjiang berries were determined. Overall, 23 polyphenols were detected in sea buckthorn, with a total polyphenol content of 634.56 mg/kg; 18 were detected in mulberry, with a total polyphenol content of 515.55 mg/kg; 17 were detected in black wolfberry, with a total polyphenol content of 324.16 mg/kg; and 12 were detected in red wolfberry, with a total polyphenol content of 299.64 mg/kg. Eight polyphenolic compounds were detected in all 4 kinds of berries, including 4-hydroxybenzoic acid, p-coumaric acid, ferulic acid, erucic acid, rutin, hypericin, kaempferol-3-O-rutinoside, and daffinoside. Six polyphenolic compounds, catechin, syringic acid, isorhamnetin-3-O-galactoside, isorhamnetin-3-O-glucoside, cinnamic acid, and isorhamnetin, were detected only in the sea buckthorn samples. The developed method has high sensitivity, accuracy, and precision and is suitable for the rapid quantitative determination of polyphenols in berry samples. This method can be used for the determination of polyphenolic compounds in berries and their products and provide data for the development of functional berry foods.

## Figures and Tables

**Table 1:** Mass spectrum parameters of 32 phenolic compounds.

Component	Precursor Ion (*m*/*z*)	Product Ion (*m*/*z*)	Fragment (V)	Collision Energy (eV)
arbutin	271.0	108.0 */160.8	115	25/7
gallic acid	169.1	125.0 */78.9	100	10/25
4-hydroxybenzoic acid	137.0	92.9 */65.0	60	10/30
epigallocatechin	305.1	125.0 */219.1	120	20/10
neochlorogenic acid	353.1	191.1 */179.0	80	10/10
D-quinic acid	191.2	85.1 */93.0	125	20/20
catechin	288.9	244.9 */202.9	130	10/15
caffeic acid	179.1	135.1 */106.9	100	15/25
p-coumaric acid	163.0	119.1 */93.0	70	15/35
syringic acid	197.0	182.0 */123.3	60	10/25
epicatechin	289.0	245.0 */203.0	125	10/15
vanillic acid	167.0	108 */152.1	50	20/10
7-hydroxycoumaric acid	161.1	133.1 */105.1	100	20/25
chlorogenic acid	366.9	135.1 */178.8	70	30/20
ferulic acid	193.1	134.0 */177.9	70	15/10
sinapic acid	223.1	208.0 */164.1	70	10/7
rutin	609.1	299.8 */270.8	105	40/60
isoferulic acid	192.9	134.2 */178.2	90	15/10
hyperoside	463.0	299.9 */270.8	170	30/50
isoquercitrin	462.9	299.8 */270.8	150	30/45
quercetin 3-O-β-D-xylopyranoside	433.1	299.9 */270.9	130	25/45
guaijaverin,	433.0	299.8 */270.9	120	25/45
salicylic acid	137.1	93.0 */65.1	80	15/35
kaempferol 3-rutinoside	593.1	285.1 */255	140	30/55
narcissin	623.0	315.0 */299.0	140	25/50
astragalin	447.1	283.9 */255	100	25/35
isorhamnetin 3-O-galactoside	477.0	313.8 */271.0	140	25/45
isorhamnetin 3-O-glucoside	477.0	314.0 */271.1	140	25/45
phloridin	435.1	272.9 */166.9	160	10/30
cinnamic acid	147.0	103.1 */77.1	70	7/25
naringin	271.1	151.0 */119.0	80	15/20
isorhamnetin	315.1	300.0 */151.0	100	20/25

* Quantitative ions.

**Table 2:** Linear ranges, linear equations, R^2^, LOD, and LOQ of 32 polyphenolic compounds.

Compound	Linear Range (μg/mL)	Linear Equation	R^2 a^	LOD ^b^ (μg/kg)	LOQ ^c^ (μg/kg)
arbutin	2–200	y = 845.1x + 777.4	0.9983	0.4	1.2
gallic acid	2–500	y = 8647.5x + 43,895.1	0.9995	0.4	1.2
4-hydroxybenzoic acid	2–500	y = 9786.5x − 823.5	0.9998	0.4	1.2
epigallocatechin	2–1000	y = 788.0x + 5422.5	0.9989	0.4	1.2
neochlorogenic acid	5–1000	Y = 30,824.5x + 6201.7	0.9977	1.0	3.0
D-quinic acid	2–1000	Y = 1934.8x + 2876.5	0.9996	0.4	1.2
catechin	2–1000	Y = 1067.3x + 16,904.7	0.9986	0.4	1.2
caffeic acid	2–500	Y = 24,638.5x + 186,859.8	0.9973	0.4	1.2
p-coumaric acid	5–1000	Y = 1163.2x − 392.0	0.9988	1.0	3.0
syringic acid	5–1000	Y = 1588.9x + 8207.0	0.9962	1.0	3.0
epicatechin	5–500	Y = 17,678.2x − 3395.0	0.9980	1.0	3.0
vanillic acid	5–1000	Y = 506.7x + 1608.1	0.9970	1.0	3.0
7-hydroxycoumaric acid	2–500	Y = 7133.8x + 24,684.8	0.9980	0.4	1.2
chlorogenic acid	5–500	Y = 4547.0x + 6755.4	0.9986	1.0	3.0
ferulic acid	1–500	Y = 1397.5x + 10,877.0	0.9966	0.2	0.6
sinapic acid	2–1000	Y = 435.4x + 714.7	0.9966	0.4	1.2
rutin	1–500	Y = 5789.4x − 1310.0	0.9976	0.2	0.6
isoferulic acid	1–500	Y = 254.6x − 153.4	0.9977	0.2	0.6
hyperoside	5–500	Y = 11,039.7x − 31,457.0	0.9961	1.0	3.0
isoquercitrin	2–500	Y = 8308.4x − 11,066.6	0.9968	0.4	1.2
quercetin 3-O-β-D-xylopyranoside	2–500	Y = 112,942.5x + 912,541.6	0.9966	0.4	1.2
guaijaverin,	2–500	Y = 57,548.4x + 456,777.7	0.9959	0.4	1.2
salicylic acid	2–1000	Y = 71,012.9x + 10,913.0	0.9957	0.4	1.2
kaempferol 3-rutinoside	5–1000	Y = 368.9x − 1140.5	0.9973	1.0	3.0
narcissin	2–1000	Y = 18,258.5x + 117,031.6	0.9994	0.4	1.2
astragalin	2–1000	Y = 8587.7x − 3507.1	0.9997	0.4	1.2
isorhamnetin 3-O-galactoside	2–1000	Y = 27,568.0x + 410,150.4	0.9967	0.4	1.2
isorhamnetin 3-O-glucoside	2–500	Y = 56,161.1x − 59,046.1	0.9994	0.4	1.2
phloridin	2–500	Y = 1522.0x + 29,246.4	0.9994	0.4	1.2
cinnamic acid	5–500	Y = 1670.4x − 4455.4	0.9974	1.0	3.0
naringin	5–500	Y = 9614.4x + 42,844.2	0.9956	1.0	3.0
isorhamnetin	5–1000	Y = 4794.5x − 10,609.5	0.9983	1.0	3.0

a: Correlation coefficients; b: limit of detection; c: limit of quantification.

**Table 3:** Recovery and RSD of 32 phenolic compounds.

Compound	Recoveries of Phenolic Compounds in Different Berries ± RSD ^a^ (%)			
	Black Wolfberry	Red Wolfberry	Mulberry	Sea Buckthorn
arbutin	88.3 ± 0.6	96.6 ± 1.5	85.6 ± 2.4	99.1 ± 2.5
gallic acid	97.5 ± 2.3	93.4 ± 2.3	97.3 ± 3.5	94.5 ± 3.2
4-hydroxybenzoic acid	89.3 ± 3.4	102.4 ± 1.8	95.5 ± 2.9	101.6 ± 3.2
epigallocatechin	95.1 ± 2.7	95.6 ± 2.8	85.6 ± 1.5	94.9 ± 1.4
neochlorogenic acid	89.9 ± 3.4	91.0 ± 2.6	97.1 ± 2.5	102.1 ± 2.4
D-quinic acid	97.5 ± 3.6	93.8 ± 3.7	90.3 ± 4.2	83.7 ± 5.2
catechin	97.9 ± 0.7	92.3 ± 4.2	100.2 ± 2.7	89.9 ± 3.9
caffeic acid	100.0 ± 3.1	93.2 ± 4.5	85.9 ± 2.9	85.1 ± 2.3
p-coumaric acid	93.3 ± 4.2	92.4 ± 3.3	90.1 ± 3.2	94.2 ± 2.8
syringic acid	100.4 ± 2.6	96.2 ± 5.1	102.2 ± 1.8	97.3 ± 1.4
epicatechin	88.7 ± 1.4	95.2 ± 3.2	92.0 ± 2.1	96.6 ± 0.4
vanillic acid	98.6 ± 2.8	90.7 ± 2.6	87.3 ± 3.1	102.4 ± 2.1
7-hydroxycoumaric acid	98.2 ± 2.6	88.5 ± 1.2	88.6 ± 2.9	98.1 ± 3.2
chlorogenic acid	93.6 ± 1.4	88.3 ± 2.2	89.9 ± 2.7	94.4 ± 2.8
ferulic acid	89.1 ± 4.5	91.4 ± 2.4	91.5 ± 4.2	86.8 ± 0.5
sinapic acid	92.6 ± 4.8	94.4 ± 2.6	90.9 ± 4.4	97.9 ± 2.9
rutin	84.9 ± 3.7	95.1 ± 2.9	96.7 ± 3.6	97.6 ± 3.8
isoferulic acid	89.6 ± 1.6	97.0 ± 2.4	99.1 ± 4.6	87.8 ± 1.2
hyperoside	84.3 ± 4.4	97.8 ± 2.7	90.1 ± 4.2	90.9 ± 2.7
isoquercitrin	88.5 ± 2.1	89.9 ± 4.2	96.4 ± 3.2	91.3 ± 4.2
quercetin 3-O-β-D-xylopyrnoside	100.4 ± 4.9	85.6 ± 3.1	95.2 ± 4.5	98.1 ± 1.9
guaijaverin,	95.4 ± 3.2	95.6 ± 4.5	98.9 ± 4.8	88.4 ± 4.7
salicylic acid	101.4 ± 3.0	104.8 ± 2.5	98.9 ± 1.8	89.6 ± 3.1
kaempferol 3-rutinoside	85.7 ± 4.2	96.7 ± 3.9	88.2 ± 3.7	95.5 ± 2.4
narcissin	87.0 ± 1.6	97.2 ± 2.9	101.9 ± 4.4	89.5 ± 1.3
astragalin	94.8 ± 5.2	90.1 ± 5.7	95.9 ± 5.8	96.6 ± 4.3
isorhamnetin 3-O-galactoside	93.6 ± 3.4	92.2 ± 3.4	99.8 ± 2.7	88.5 ± 4.6
isorhamnetin 3-O-glucoside	97.3 ± 1.4	96.4 ± 4.1	85.6 ± 1.7	82.8 ± 2.7
phloridin	97.3 ± 0.7	90.9 ± 3.9	88.5 ± 4.5	95.3 ± 3.2
cinnamic acid	86.6 ± 3.1	92.6 ± 4.1	94.2 ± 2.5	92.6 ± 1.2
naringin	104.3 ± 4.1	103.1 ± 3.9	86.5 ± 4.8	90.2 ± 0.5
isorhamnetin	94.3 ± 2.5	90.5 ± 5.5	91.2 ± 2.3	95.8 ± 2.9

a: Relative standard deviations.

**Table 4:** The results of actual sample determination.

Compound	The Amount of Phenolic Compounds in Different Berries (mg/kg)			
	Black Wolfberry	Red Wolfberry	Mulberry	Sea Buckthorn
gallic acid	2.11	ND	2.01	6.25
4-hydroxybenzoic acid	13.4	4.40	2.60	1.21
D-quinic acid	82.5	ND	44.4	4.71
catechin	ND	ND	ND	4.33
caffeic acid	1.60	1.19	0.96	ND
syringic acid	ND	ND	ND	0.30
epicatechin	13.5	65.9	1.64	1.98
vanillic acid	ND	ND	0.11	0.16
chlorogenic acid	55.0	3.79	25.6	ND
ferulic acid	3.81	7.89	0.35	0.54
sinapic acid	1.29	1.01	0.54	9.84
rutin	84.6	130	221	188
isoferulic acid	1.54	42.0	ND	0.18
hyperoside	1.15	0.57	95.3	169
isoquercitrin	0.42	ND	5.06	3.68
salicylic acid	0.17	0.40	ND	0.33
kaempferol 3-rutinoside	58.1	40.8	106	68.3
narcissin	2.53	1.69	0.11	117
astragalin	0.11	ND	7.15	4.69
isorhamnetin 3-O-galactoside	ND	ND	ND	3.35
isorhamnetin 3-O-glucoside	ND	ND	ND	41.4
phloridin	2.14	ND	2.59	0.26
cinnamic acid	ND	ND	ND	0.61
naringin	0.19	ND	0.13	0.11
isorhamnetin	ND	ND	ND	8.33

ND: Not detected.

**Table 5:** Compared with the methods reported in the literature.

Analytical Method	Species	LOD ^a^	LOQ ^b^	Reference
HPLC-MS/MS	32	0.2–1.0 μg/kg	0.6–3.0 μg/kg	this research
HPLC-DAD	24	0.002–0.16 μg/mL	0.01–0.48 μg/mL	[32]
HPLC-DAD	17	0.003–0.063 μg/mL	0.009–0.211 μg/mL	[33]
HPLC-DAD	16	0.03–0.62 mg/L	0.11–2.08 mg/L	[27]
HPLC-DAD	13	0.1–0.3 μg/mL	0.2–1.0 μg/mL	[34]
HPLC-DAD	10	0.016–0.144 μg/mL	0.051–0.711 μg/mL	[35]
HPLC-DAD	10	0.09–0.35 μg/mL	0.29–0.1.07 μg/mL	[36]
UPLC-DAD	41	0.10–0.65 mg/L	0.23–1.12 mg/L	[37]
UPLC-DAD	9	0.18–1.01 μg/mL	0.54–3.06 μg/mL	[38]
UPLC-PDA	7	0.02–0.17 μg/mL	0.1–0.6 μg/mL	[39]
GC-MS	23	2.5–25 ng/mL	5–50 ng/mL	[40]
GC-MS/MS	8	7.9–310 μg/kg	26–1800 μg/kg	[41]
LC-MS/MS	36	0.0004–0.0037 μg/L	0.0012–0.0111 μg/L	[26]
LC-MS/MS	32	7.5–158.3 ng/mL	22.6–479.8 ng/mL	[42]
LC-MS/MS	8	0.003–0.445 mg/L	0.010–1.483 mg/L	[23]
UPLC-Q-Orbitrap MS	25	1.05–4.59 μg/L	3.59–15.98 μg/L	[43]
HPLC-ESI-Q-Tof	13	0.01–0.15 µg/g	0.02–0.50 µg/g	[44]
UPLC- ESI-Q-Tof	4	0.01–0.12 μg/mL	0.06–0.49 μg/mL	[45]

a: Limit of detection; b: limit of quantification.

## Data Availability

Data available on request from the authors.

## Supplementary Materials

The following supporting information can be downloaded at: https://www.mdpi.com/article/10.3390/molecules30092008/s1, Figure S1: Mass spectra of 32 phenolic compounds in negative ion mode by HPLC-MS/MS, Figure S2: Determination of 32 phenolic compounds in 4 kinds of berries by HPLC-MS/MS A, Black wolfberry; B, Sea buckthorn; C, Mulberry; D, Red wolfberry.
