# Supplementary material for: Simultaneous Determination of 32 Polyphenolic Compounds in Berries via HPLC–MS/MS
Source: Molecules. 2025 Apr 30;30(9):2008. doi: 10.3390/molecules30092008 (PMC12073267; doi:10.3390/molecules30092008)
Supplement: Supplementary file 1 [file molecules-30-02008-s001.zip › Supplementary material.pdf]

Figure S1. Mass spectra of 32 phenolic compounds in negative ion mode by HPLC-MS/MS

Figure S2. Determination of 32 phenolic compounds in 4 kinds of berries by HPLC-MS/MS A,Black wolfberry; B,Sea buckthorn; C,Mulberry; D,Red wolfberry

1 arbutin

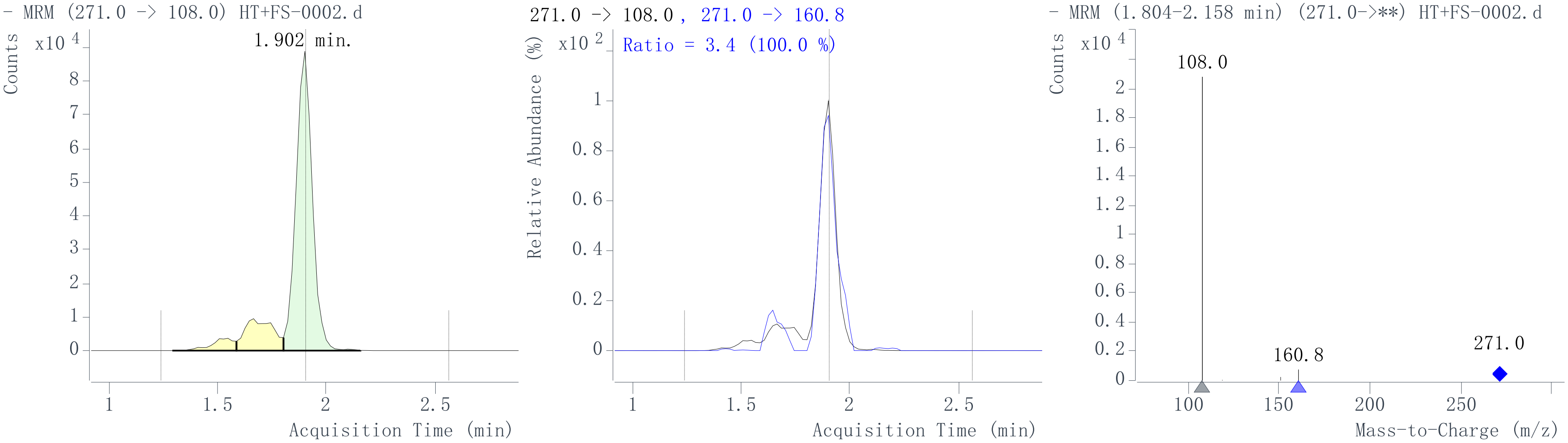

2 gallic acid

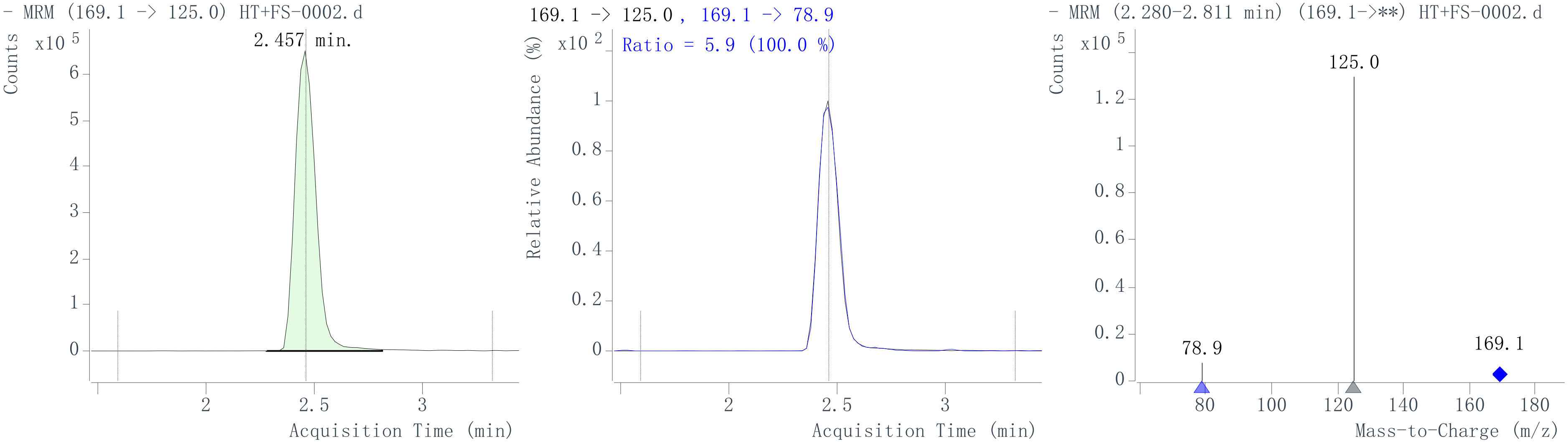

3 4-hydroxybenzoic acid

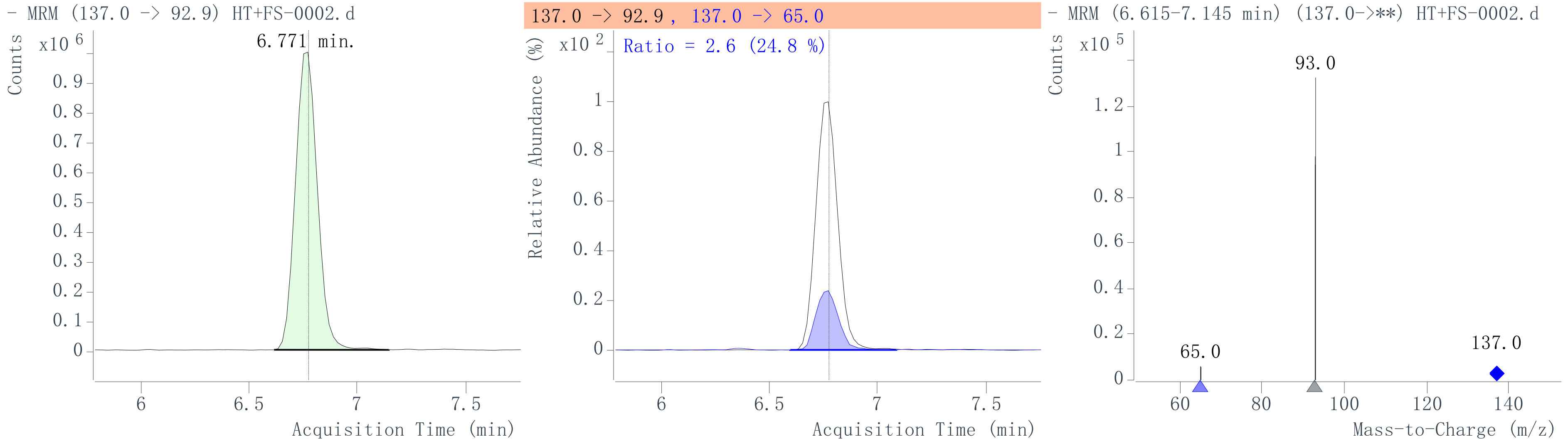

4 epigallocatechin

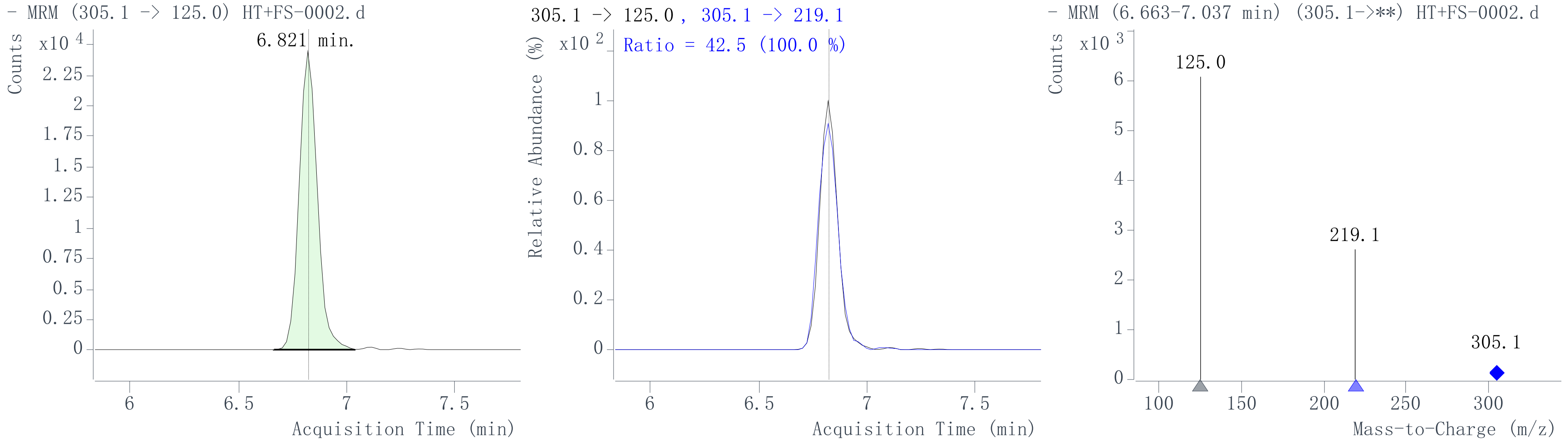

5 neochlorogenic acid

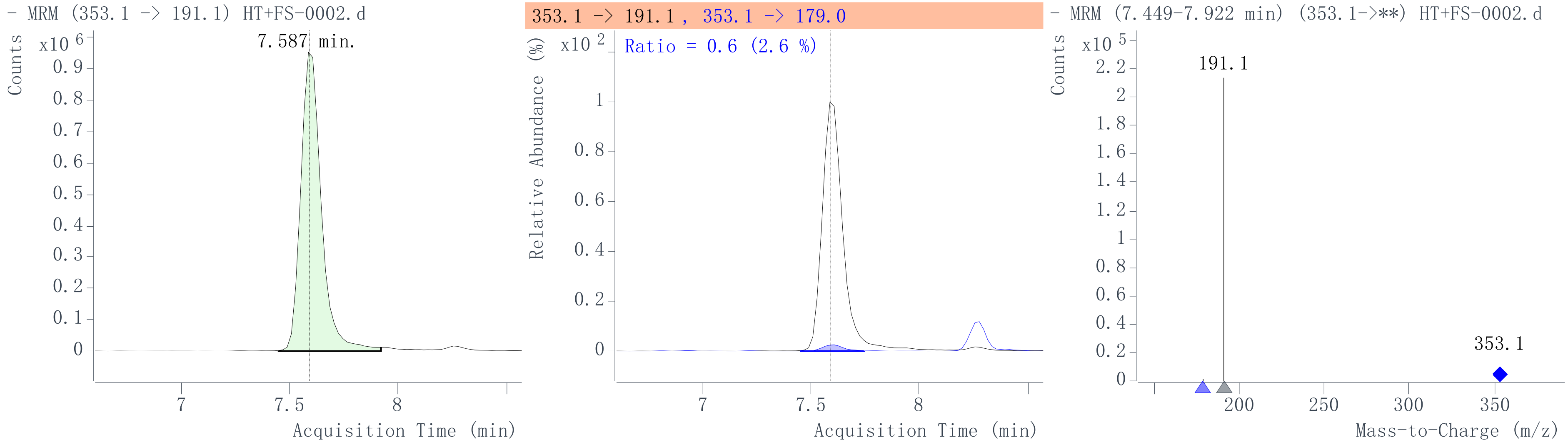

6 D-quinic acid

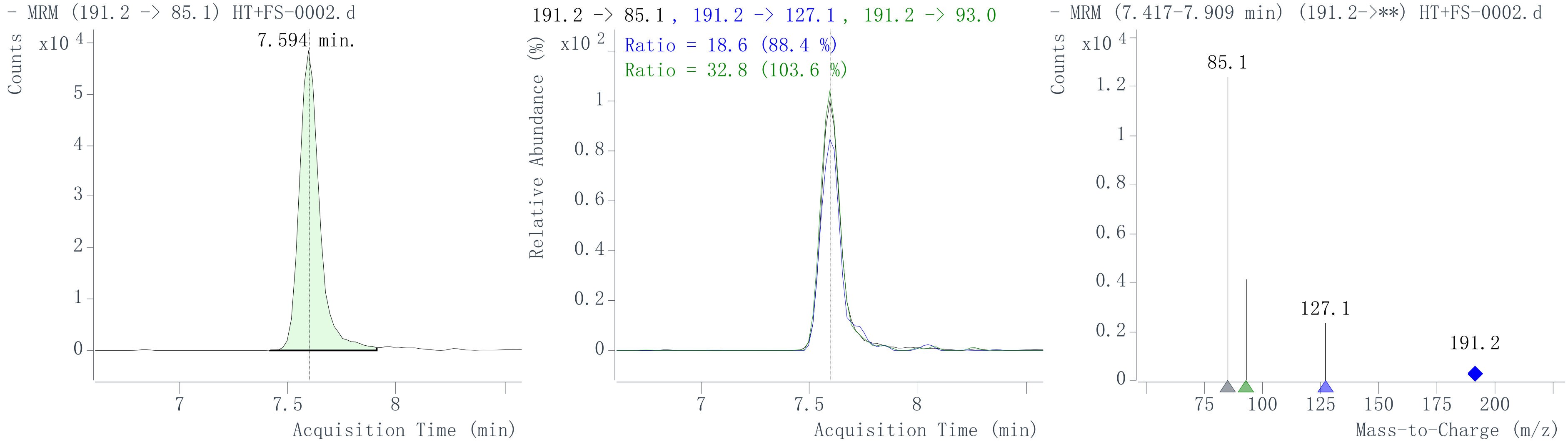

7 catechin

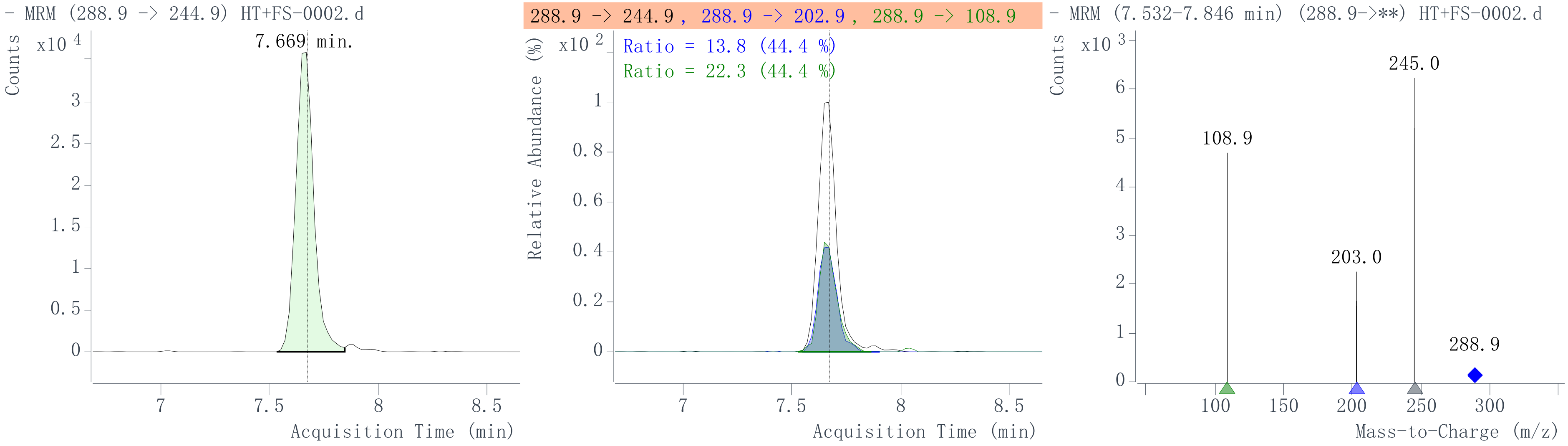

8 caffeic acid

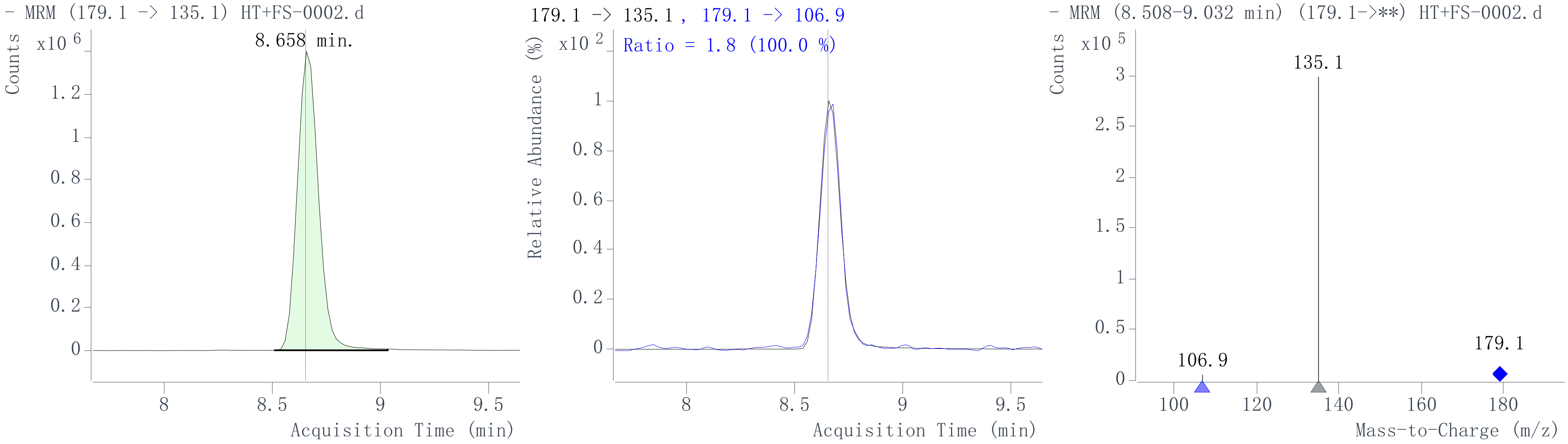

9 p-coumaric acid

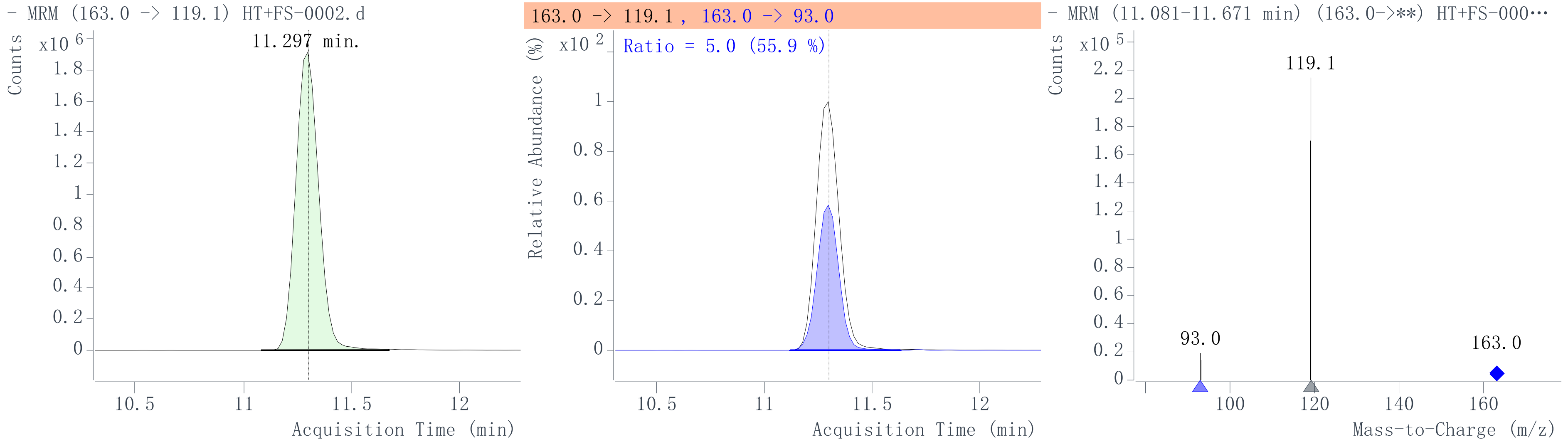

10 syringic acid

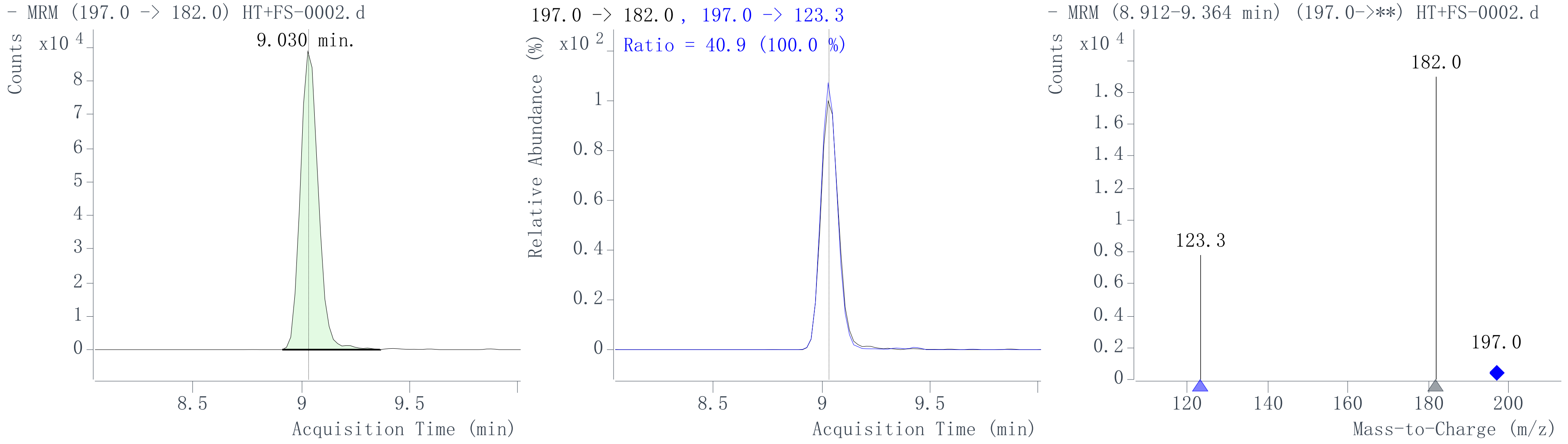

11 epicatechin

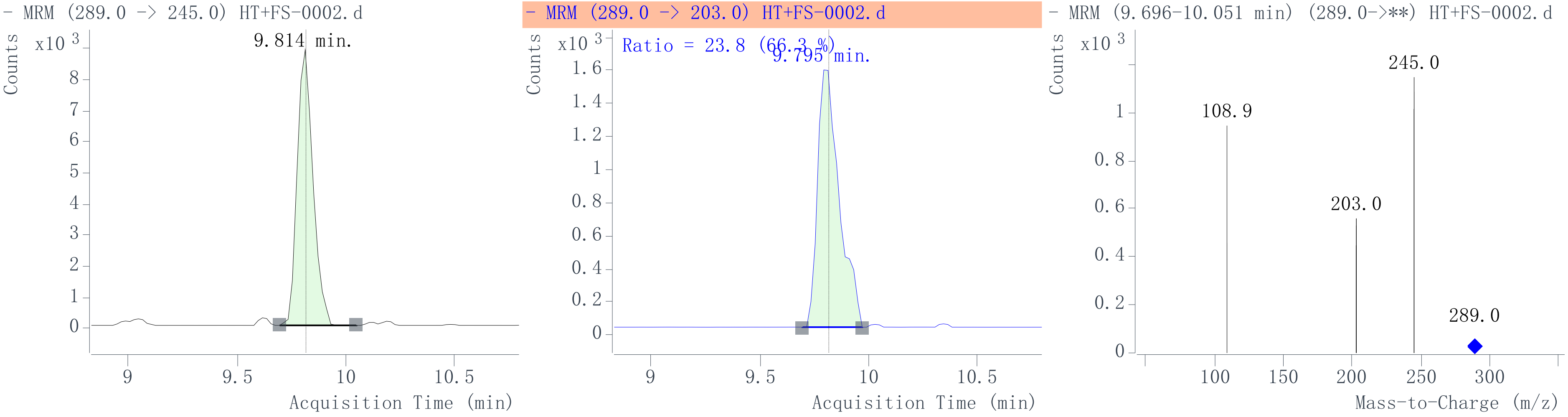

12 vanilloid acid

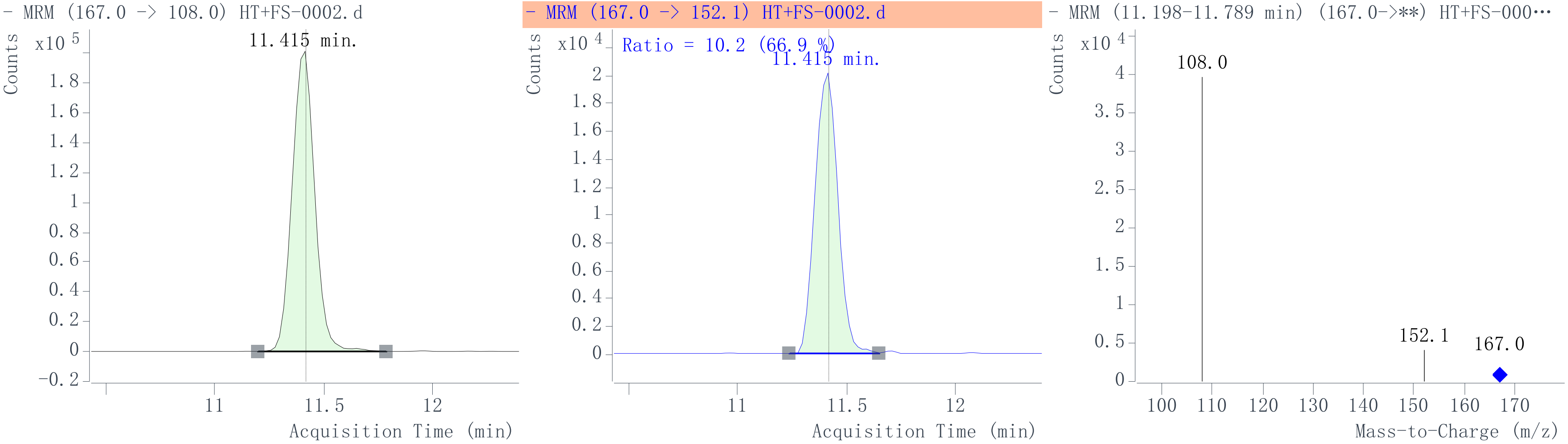

13 7-hydroxycoumaric acid

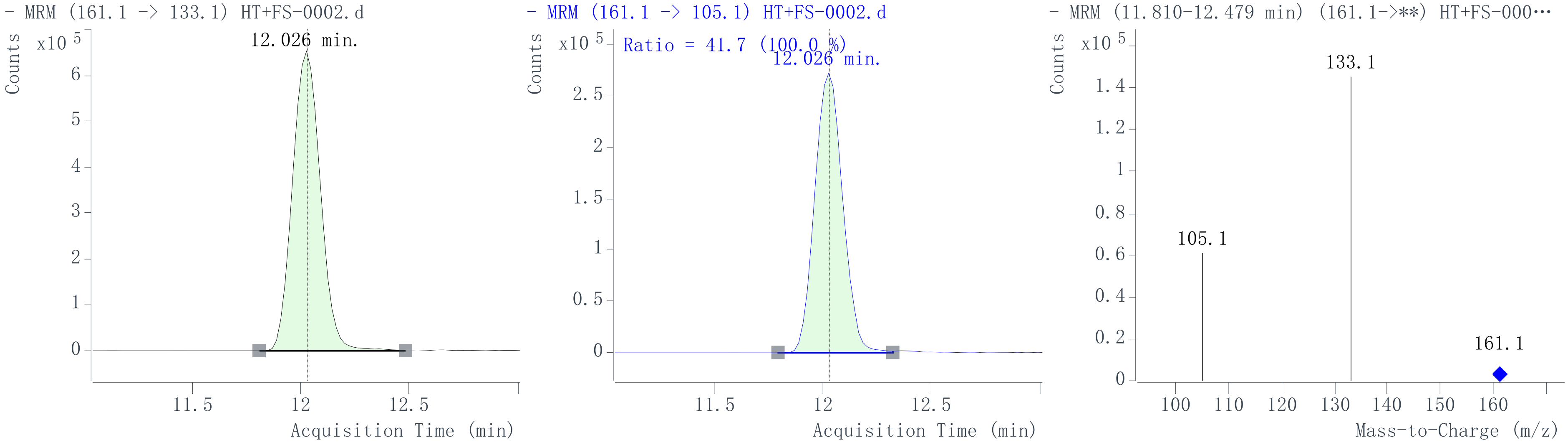

14 chlorogenic acid

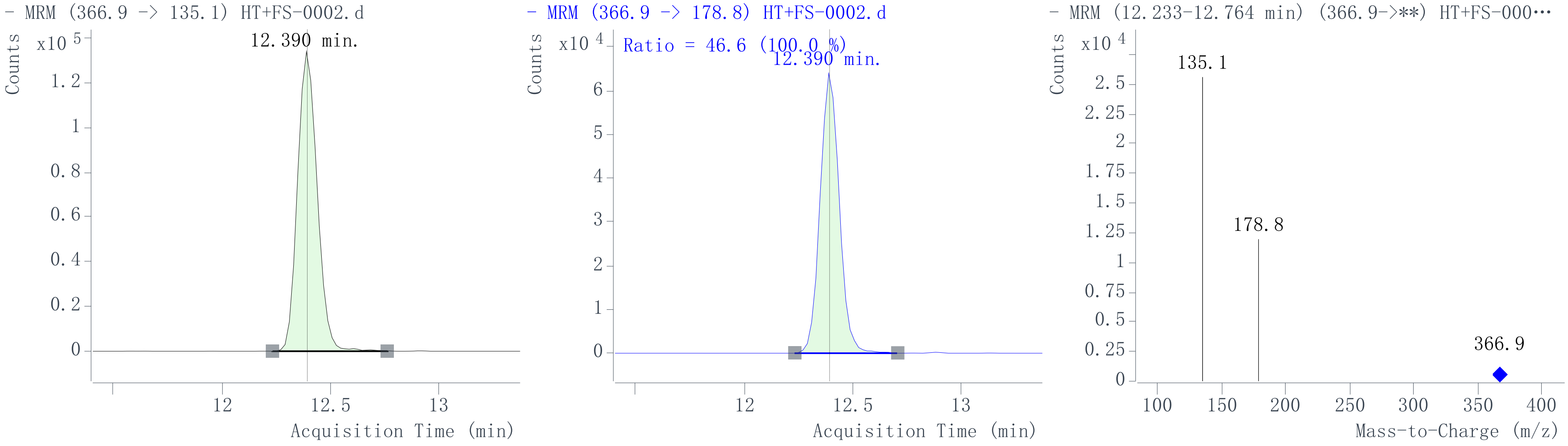

15 ferulic acid

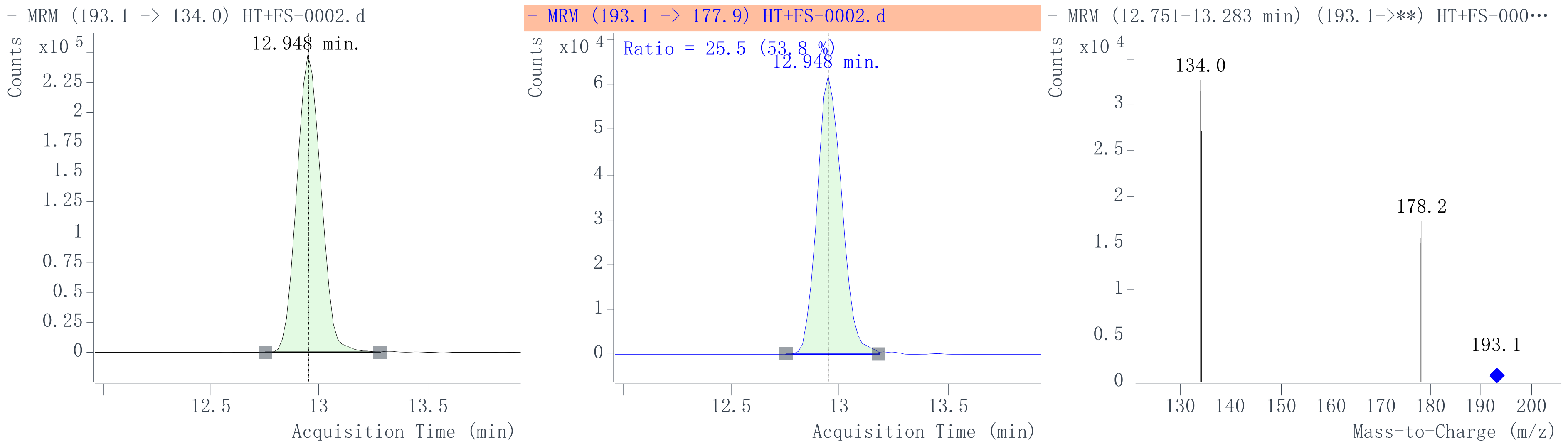

16 sinapic acid

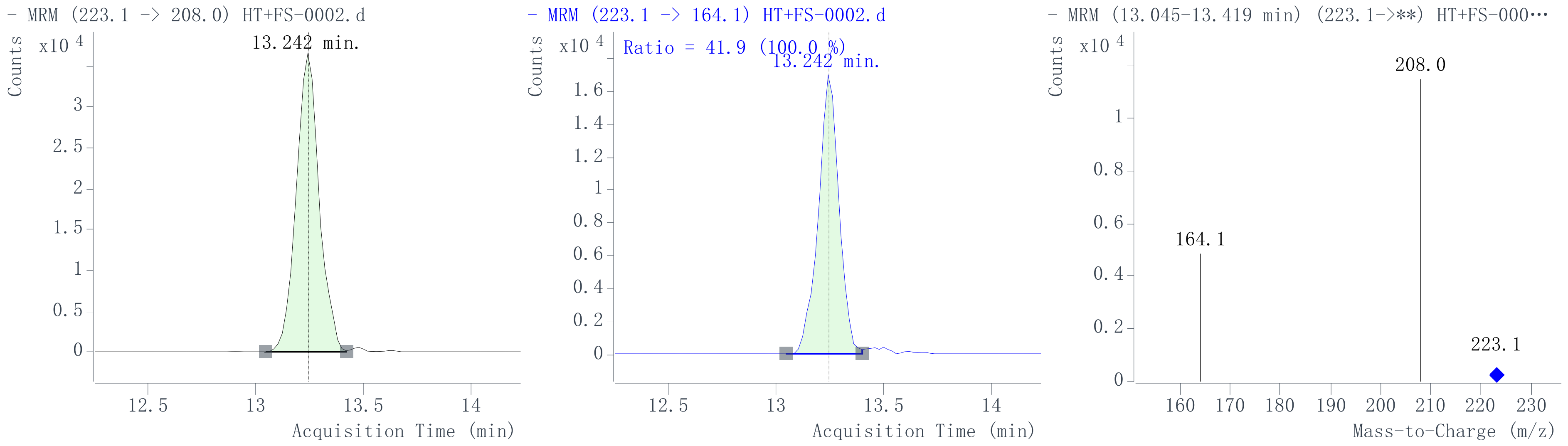

17 rutin

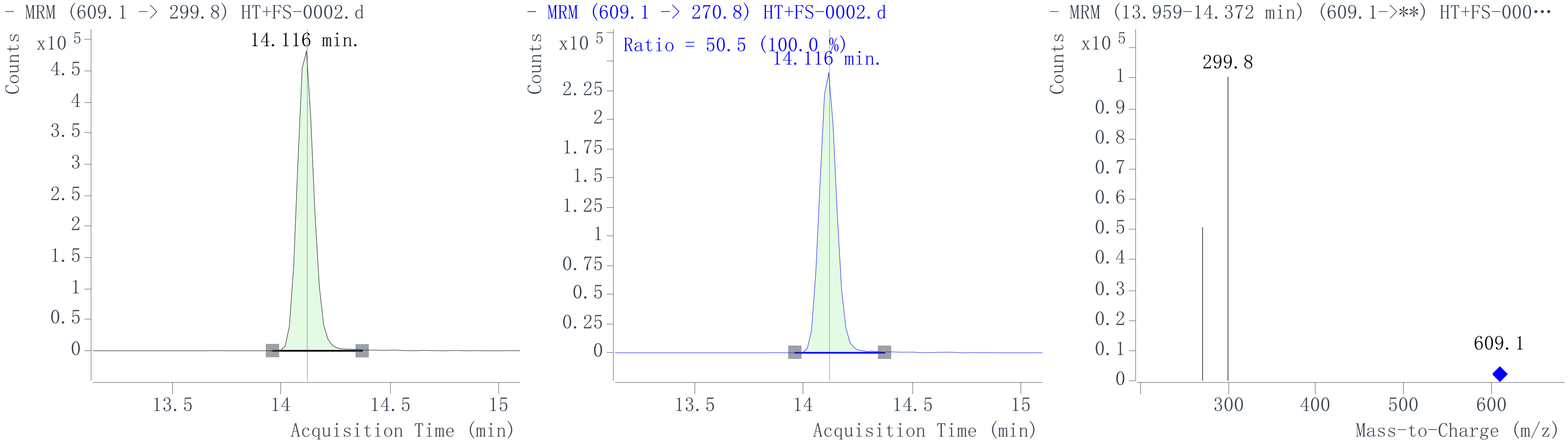

18 isoferulic acid

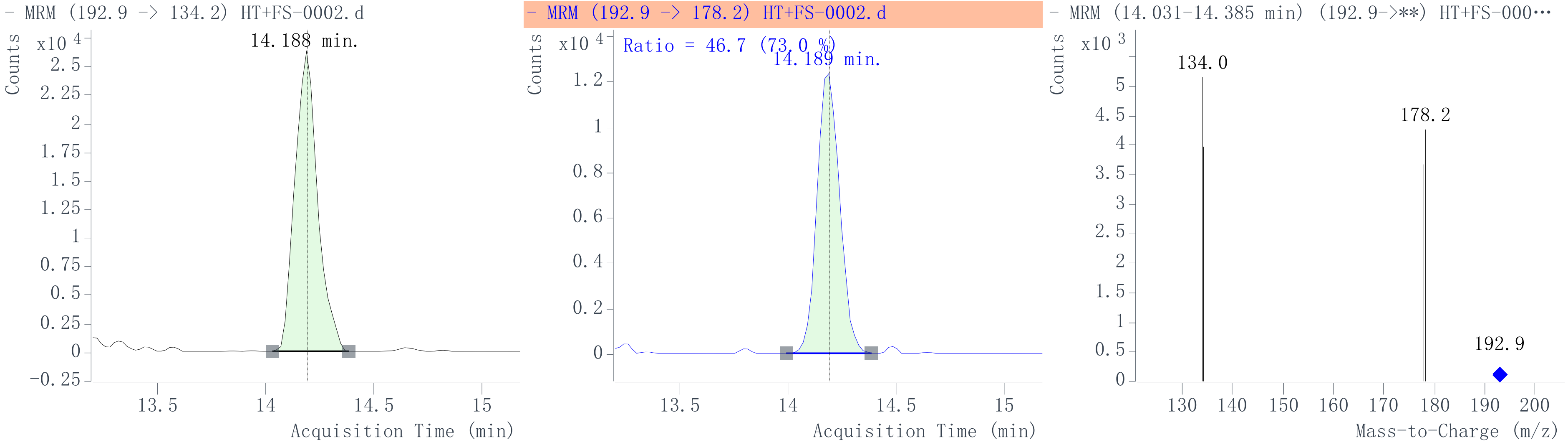

19 hyperoside

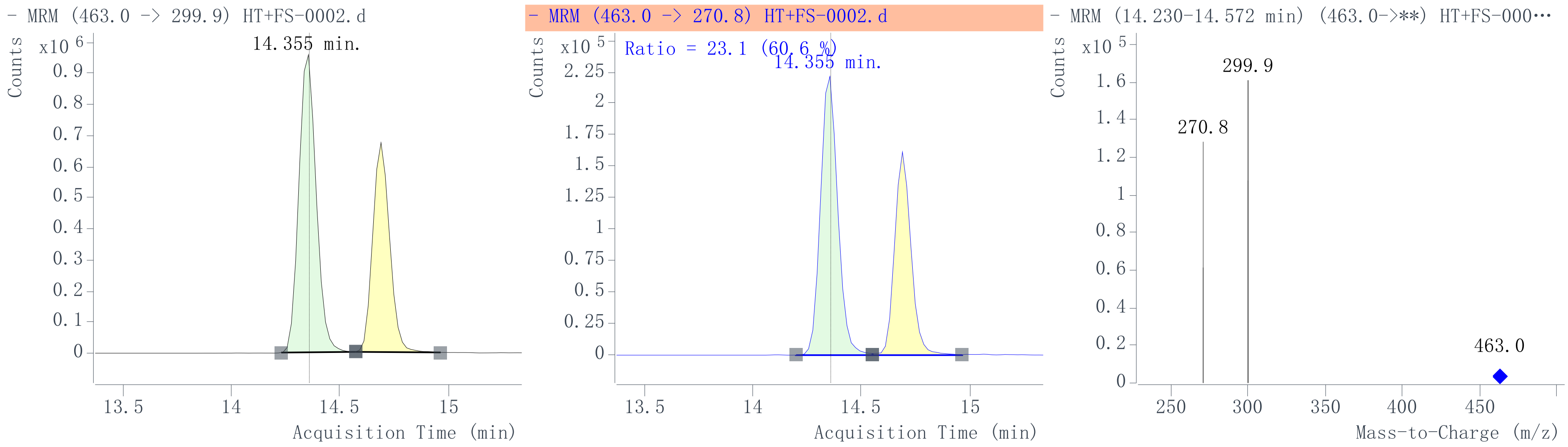

20 isoquercitrin

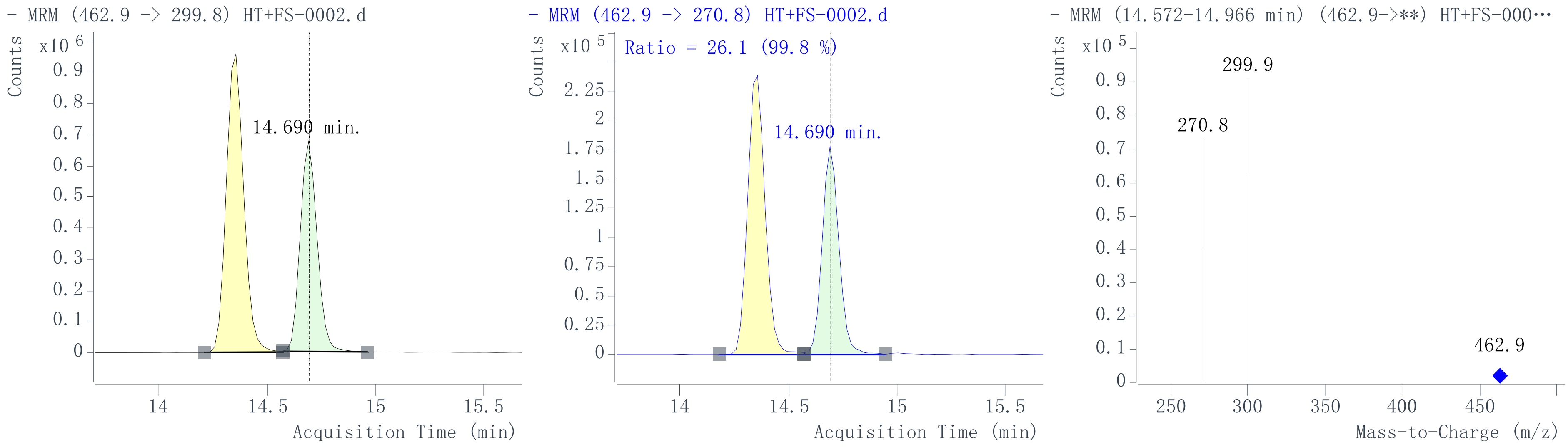

21 3-O-β-D-xylopyranoside

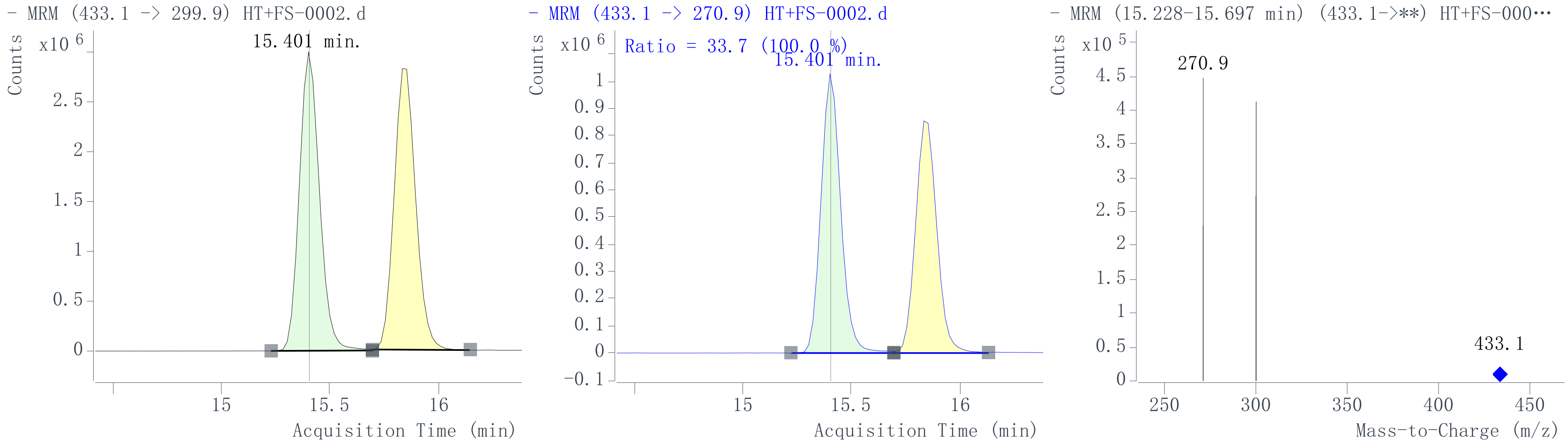

22 guaijaverin

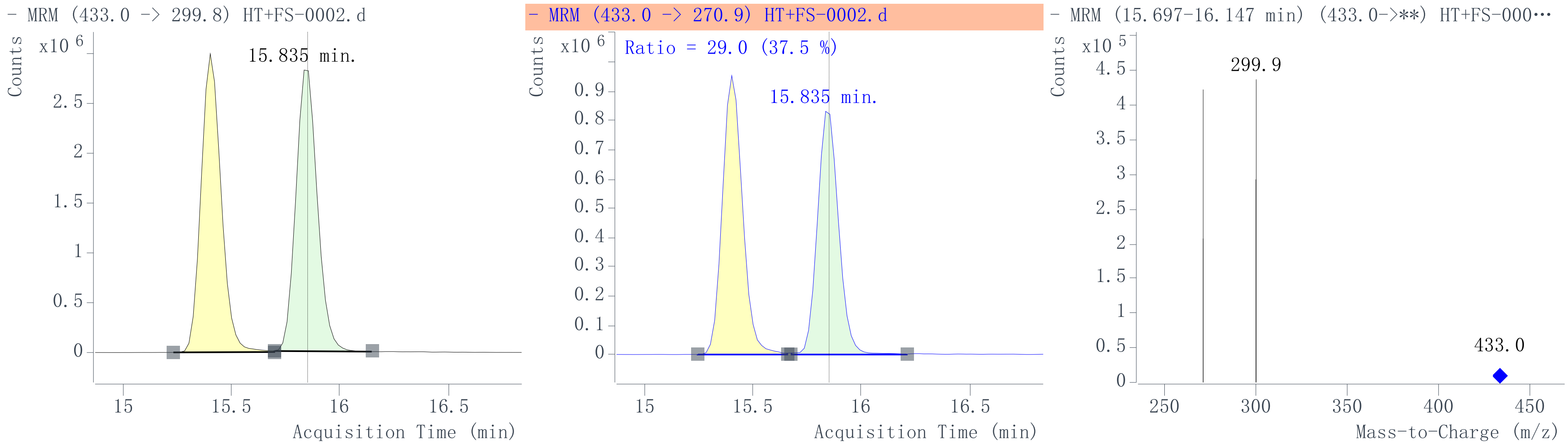

23 salicylic acid

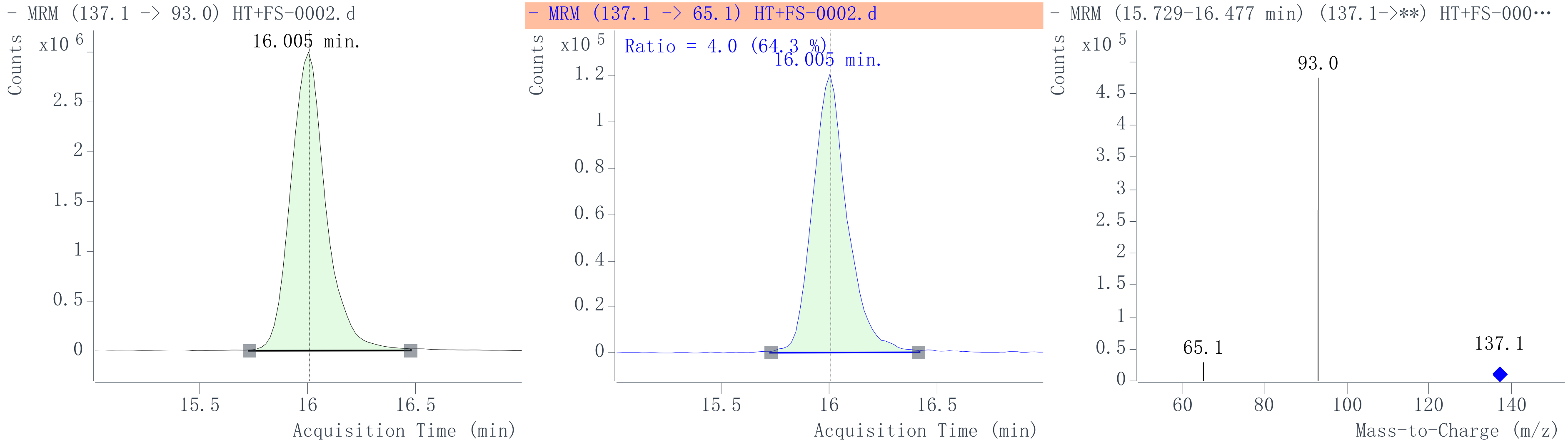

24 kaemoferol 3-rutinoside

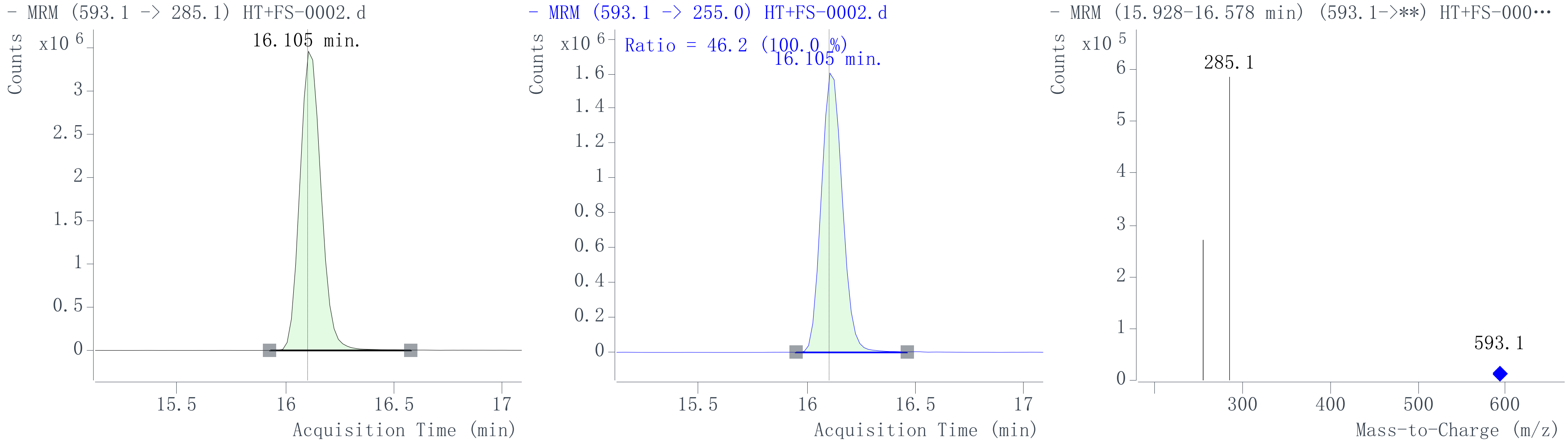

25 narcissin

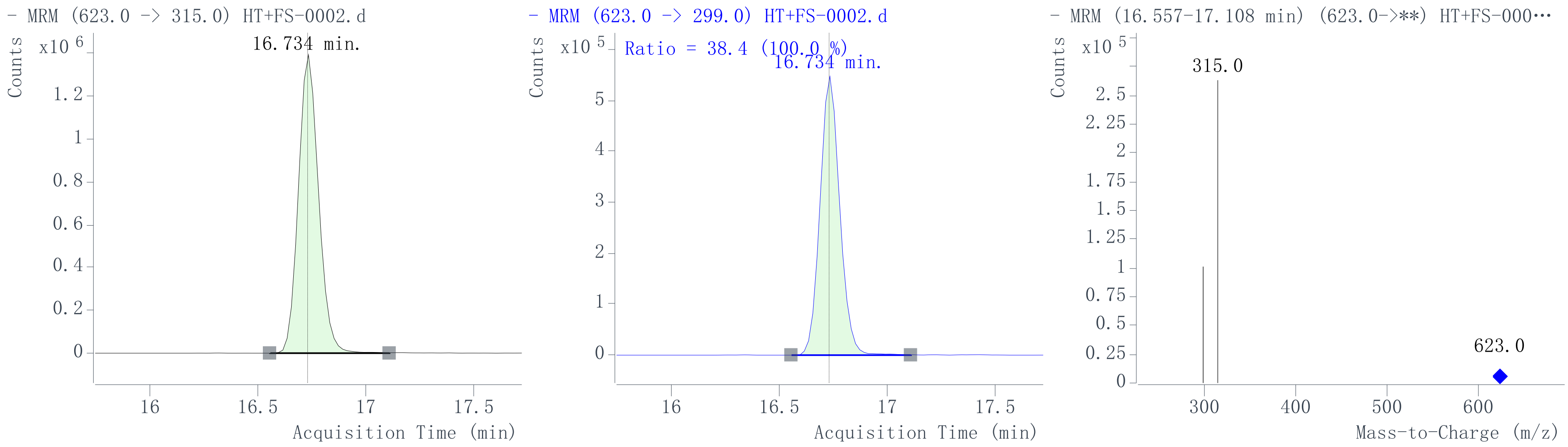

26 astragalin

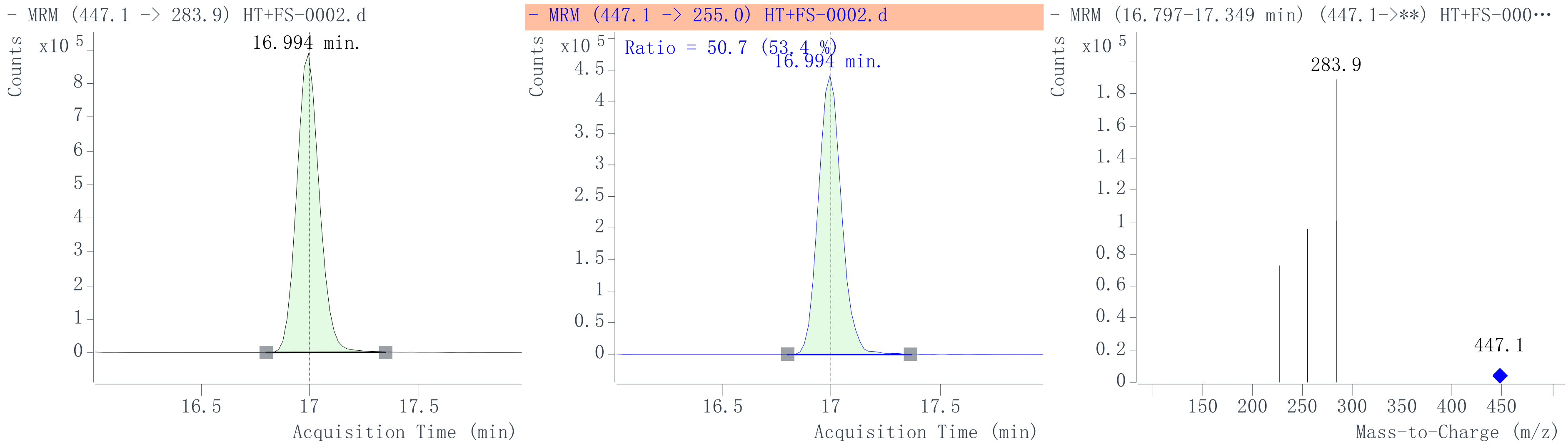

27 isorhamnetin 3-O-galactoside

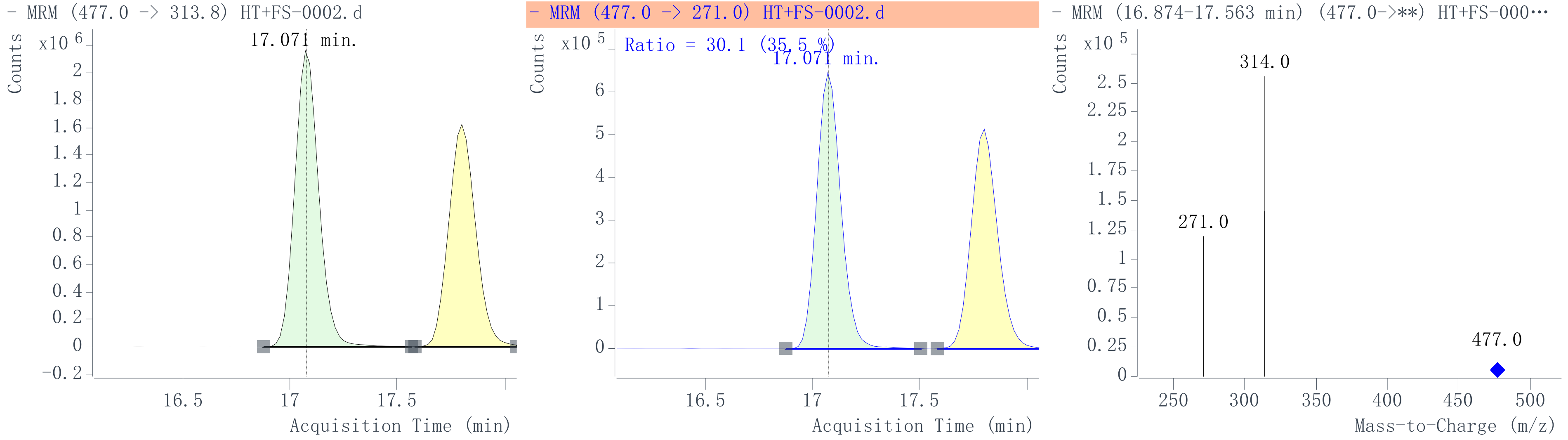

28 isorhamnetin 3-O-glucoside

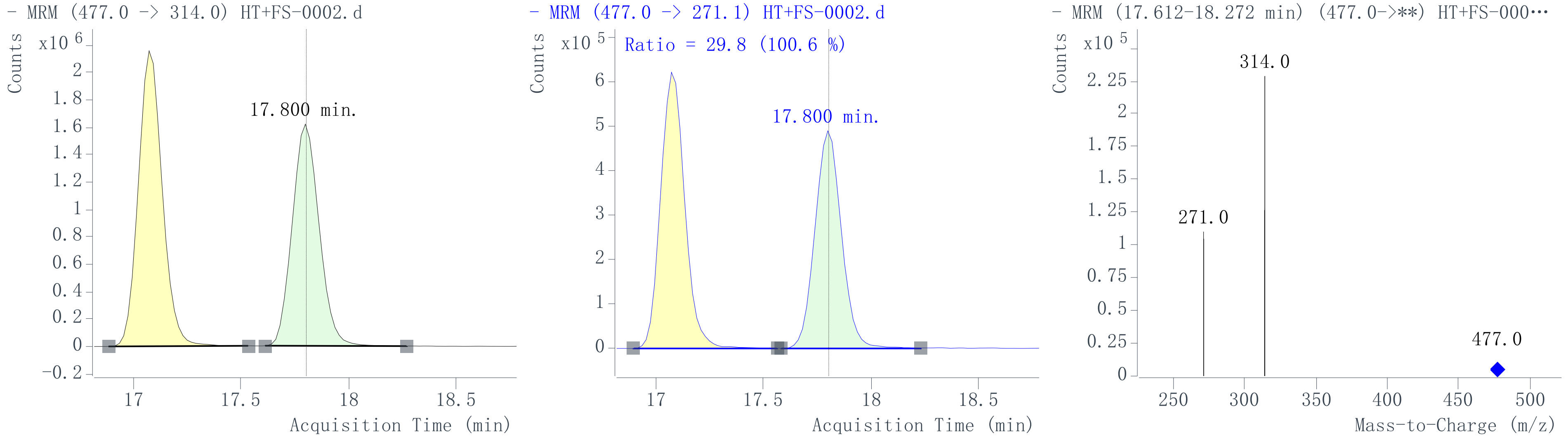

29 phloridin

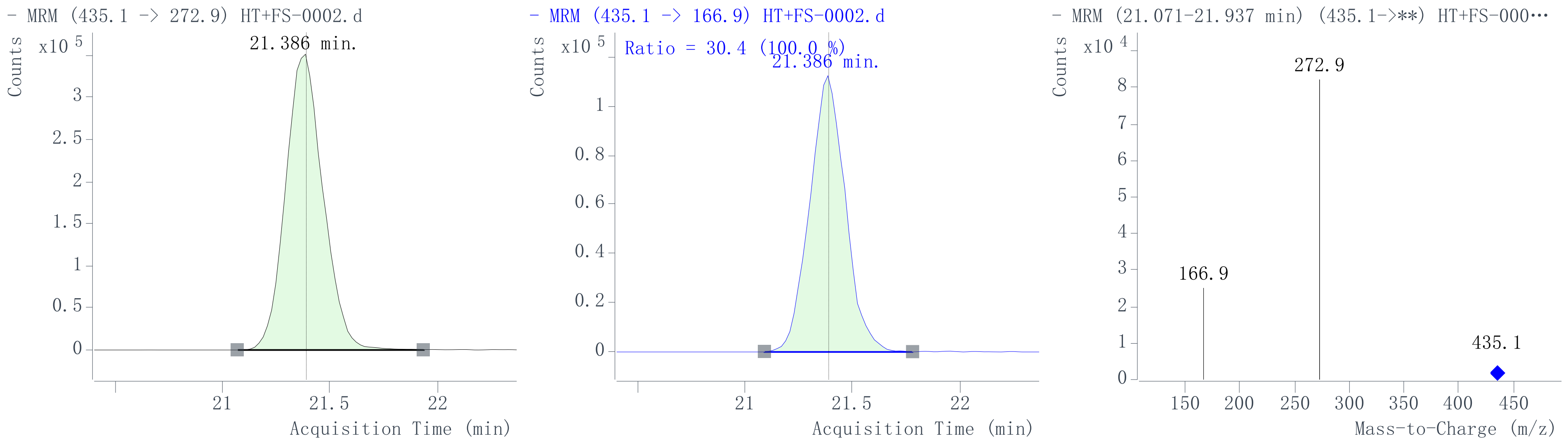

30 cinnamic acid

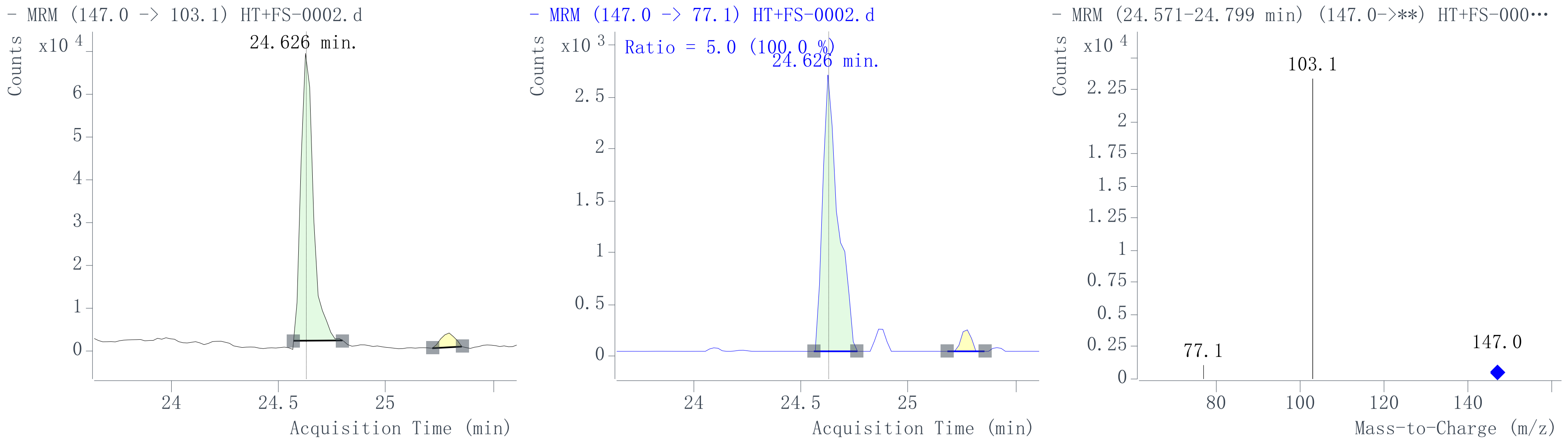

31 naringin

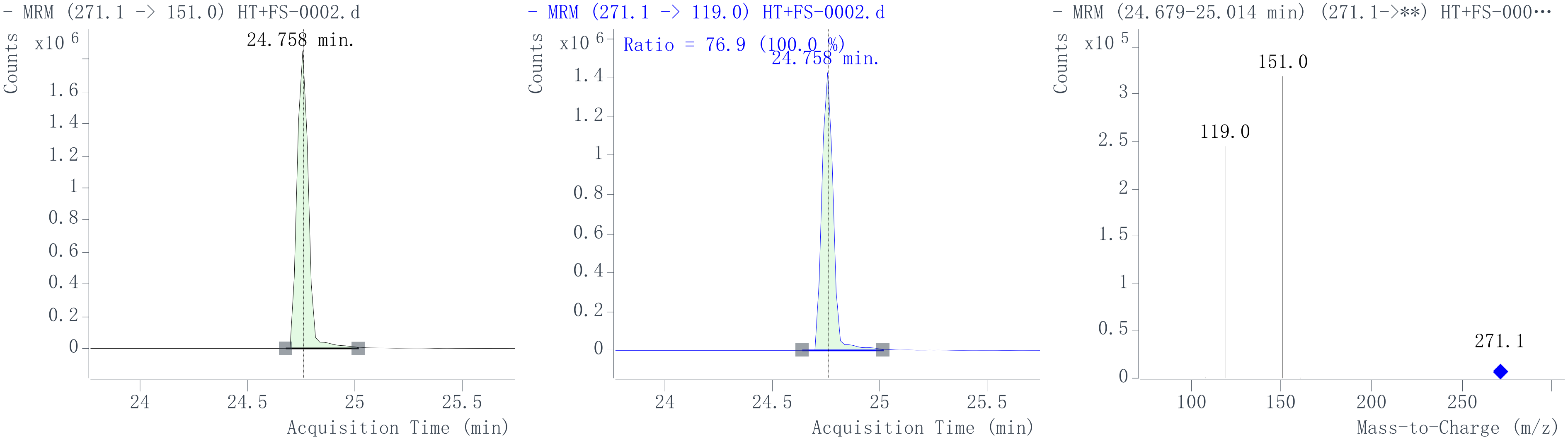

32 isorhamnetin

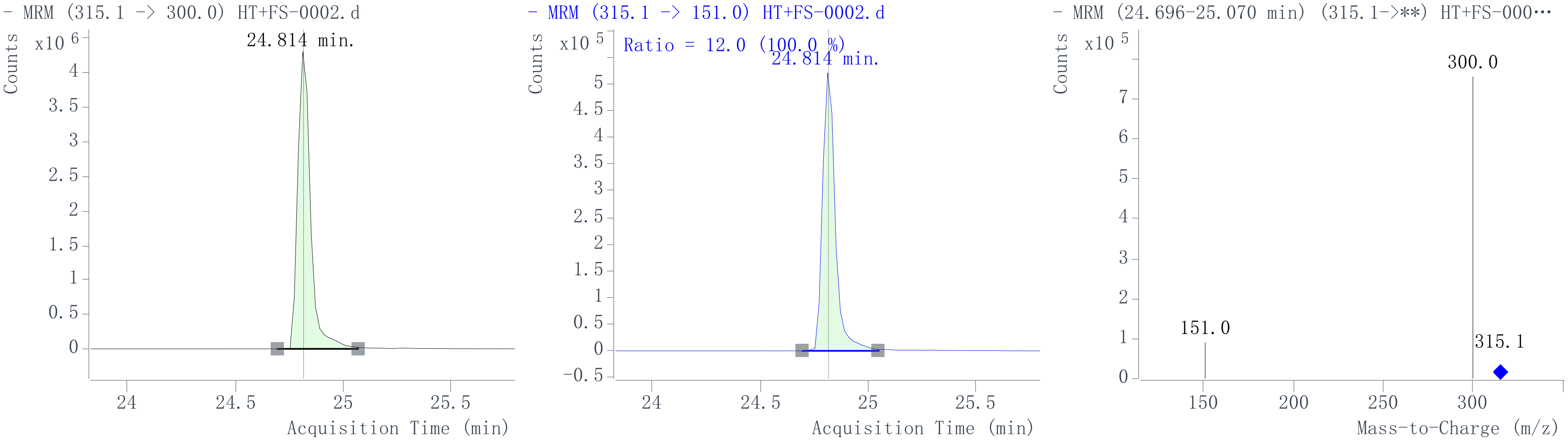

Figure S1. Mass spectra of 32 phenolic compounds in negative ion mode by HPLC-MS/MS

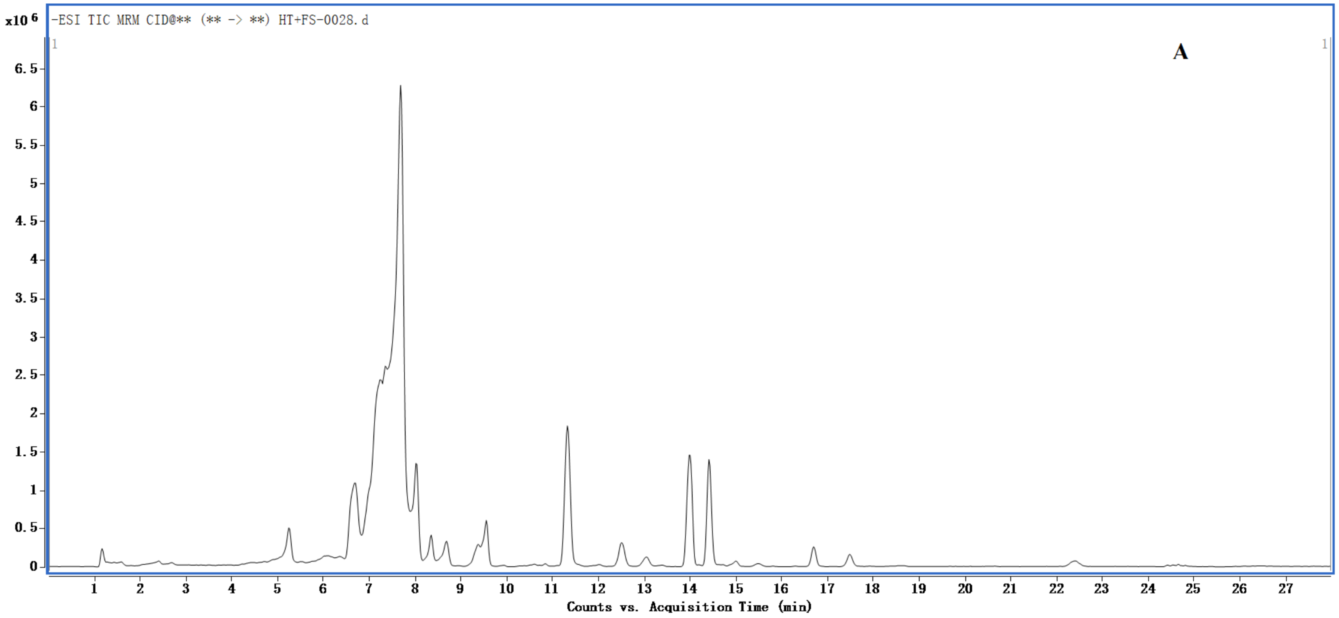
x10 6
-ESI TIC MRM CID@** (** -> **) HT+FS-0028.d
A
Counts vs. Acquisition Time (min)

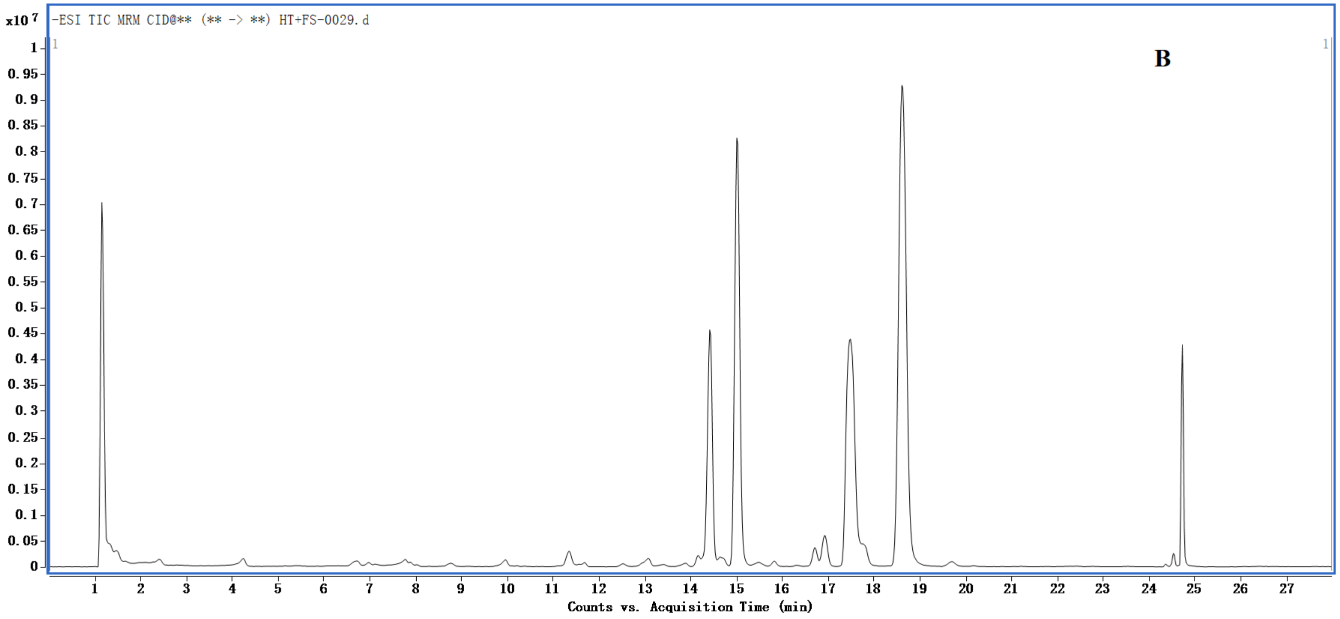
x10 7
-ESI TIC MRM CID@** (** -> **) HT+FS-0029.d
B
Counts vs. Acquisition Time (min)

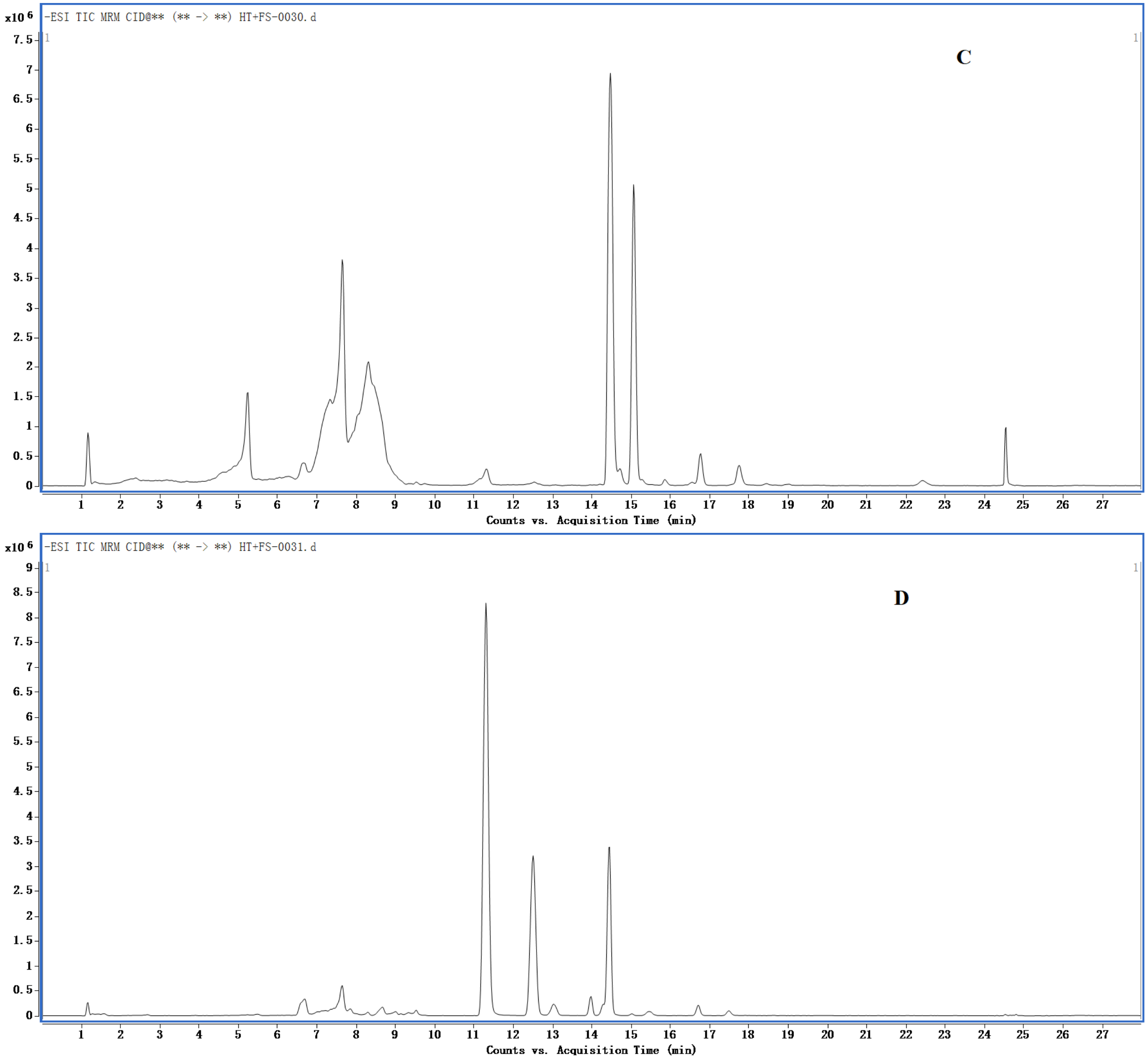

Figure S2. Determination of 32 phenolic compounds in 4 kinds of berries by HPLC-MS/MS

A,Black wolfberry; B,Sea buckthorn; C,Mulberry; D,Red wolfberry
